# Targeting Deltex E3 Ubiquitin Ligase 2 Inhibits Tumor‐associated Neutrophils and Sensitizes Hepatocellular Carcinoma Cells to Immunotherapy

**DOI:** 10.1002/advs.202408233

**Published:** 2024-12-29

**Authors:** Xiaoling Wu, Jiafeng Chen, Yiran Chen, Shushu Song, Yuan Fang, Shengwei Mao, Jun Gao, Guiqi Zhu, Weifeng Qu, Qianfu Zhao, Rui Yang, Zhiqi Guan, Tianhao Chu, Yichao Bu, Yi Wang, Fangyu Chen, Jian Zhou, Jia Fan, Zheng Tang, Weiren Liu, Yuanyuan Ruan, Yinghong Shi

**Affiliations:** ^1^ Department of Liver Surgery and Transplantation Zhongshan Hospital Fudan University Shanghai 200032 China; ^2^ Research Unit of Liver Cancer Recurrence and Metastasis Chinese Academy of Medical Sciences Beijing 100010 China; ^3^ Key Laboratory of Carcinogenesis and Cancer Invasion of Ministry of Education Shanghai 200032 China; ^4^ Liver Cancer Institute Zhongshan Hospital Fudan University Shanghai 200032 China; ^5^ Department of Biochemistry and Molecular Biology School of Basic Medical Sciences Fudan University Shanghai 200032 China; ^6^ Department of Radiation Oncology Zhongshan Hospital Affiliated to Fudan University Shanghai 200032 China; ^7^ Institutes of Biomedical Sciences Fudan University Shanghai 200032 China; ^8^ Shanghai Key Laboratory of Organ Transplantation Shanghai 200032 China; ^9^ State Key Laboratory of Genetic Engineering and Collaborative Innovation Center for Genetics and Development School of Life Sciences Fudan University Shanghai 200032 China

**Keywords:** deltex E3 ubiquitin ligase 2, DTX2 inhibitor, hepatocellular carcinoma, tumor‐associated neutrophils

## Abstract

Several E3 ligases have been found to affect the immune microenvironment of hepatocellular carcinoma (HCC) and lead to the resistance of immunotherapy. In this study, genes of E3 ligases are screened based on The Cancer Genome Atlas (TCGA) dataset. Through cytometry by time of flight (CyTOF), flow cytometry, and further experiments, Deltex E3 ubiquitin ligase 2 (DTX2) in HCC cells is identified to promote the infiltration and polarization of tumor‐associated neutrophils (TANs) with a protumor phenotype, thus attenuating the infiltration and cytotoxicity of CD8+ T cells partially through C‐X‐C motif chemokine 2 (CXCL2) and C‐X‐C motif chemokine 6 (CXCL6). Mechanistically, DTX2 can interact with histone H2B and promote its monoubiquitination at lysine120 (H2BK120ub1), thereby increasing CXCL2 and CXCL6 transcription through histone epigenetic regulation. Different tumor models in vivo demonstrated that DTX2 inhibitor treatment inhibited tumor growth and sensitized HCC cells to the therapeutic effects of programmed cell death protein 1 (PD‐1) antibody. In summary, this study identifies DTX2 as a potential target for HCC immunotherapy.

## Graphical abstract

Several E3 ligases can affect the immune microenvironment of hepatocellular carcinoma (HCC). In this study, DTX2 in HCC cells is identified to promote the infiltration and polarization of TANs with a protumor phenotype, thus attenuating the cytotoxicity of CD8+ T cells. In summary, this study identifies DTX2 as a potential target for HCC immunotherapy.

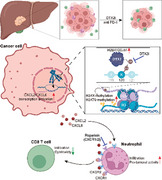

## 1 Introduction

In the microenvironment of hepatocellular carcinoma (HCC), neutrophils can gradually differentiate into tumor‐associated neutrophils (TANs) with an antitumor phenotype or a protumor phenotype.^[^ ^1^ ^]^ TANs with the protumor phenotype exhibit high expression of programmed cell death 1 ligand 1 (PD‐L1) and secrete arginase‐1 (ARG1), reactive oxygen species (ROS), and inducible nitric oxide synthase (iNOS) to suppress the tumoricidal function of effector T cells and reduce the therapeutic response to immune checkpoint inhibitors (ICIs).^[^ ^2^ ^]^ Therefore, targeting TANs or blocking polarization toward the protumor phenotype increases the efficacy of immunotherapy.

Ubiquitination relies on ubiquitin, ubiquitin‐activating (E1), ubiquitin‐conjugating (E2) and ubiquitin ligase (E3) enzymes.^[^ ^3^ ^]^ Ubiquitination of histone H2A and histone H2B mainly affect gene transcription and DNA damage repair.^[^ ^4^ ^]^ In particular, the crosstalk between monoubiquitination of H2B and polymethylation of H3 explains how monoubiquitination of histone H2B regulates gene transcription at the epigenetic level.^[^ ^5^, ^6^ ^]^

Recently, several studies suggest that the inhibition of specific ubiquitin ligases may be beneficial for the response to immunotherapy.^[^ ^7^, ^8^ ^]^ Deltex E3 ubiquitin ligase 2 (DTX2) can recognize and promote the ubiquitination of proteins with polyadenosine diphosphate ribosylation (poly‐ADP‐ribosylation, PARylation) through its DTC domain.^[^ ^9^ ^]^ In addition, DTX2 participates in different types of ubiquitination modifications.^[^ ^10^, ^11^ ^]^ However, the specific role of DTX2 in the tumor immune microenvironment remains unknown.

Based on analysis of liver hepatocellular carcinoma (LIHC) RNA sequencing (RNA‐seq) data in TCGA, we screened for genes encoding E3 ligases and revealed that DTX2 in tumor cells might be related to immune cell infiltration in HCC tissues. Subsequently, we found that DTX2 promoted the secretion of CXCL2 and CXCL6 from HCC cells to recruit neutrophils and polarize them toward a protumor phenotype, thus attenuating the proliferation and cytotoxicity of CD8+ T cells. Mechanistically, DTX2 promoted monoubiquitination of histone H2B at lysine 120 (H2BK120ub1), which altered chromatin accessibility and affected the transcription of C‐X‐C motif chemokine 2 (CXCL2) and C‐X‐C motif chemokine 6 (CXCL6). Targeting DTX2 decreased the related histone epigenetic modifications of CXCL2 and CXCL6, thereby attenuating the immunosuppressive characteristics of the TME and sensitizing HCC cells to anti‐PD1 immunotherapy.

## 2 Results

### 2.1 DTX2 in HCC Cells Promotes Tumor Growth by Affecting TANs and CD8+ T Cells

**Figure 1:**
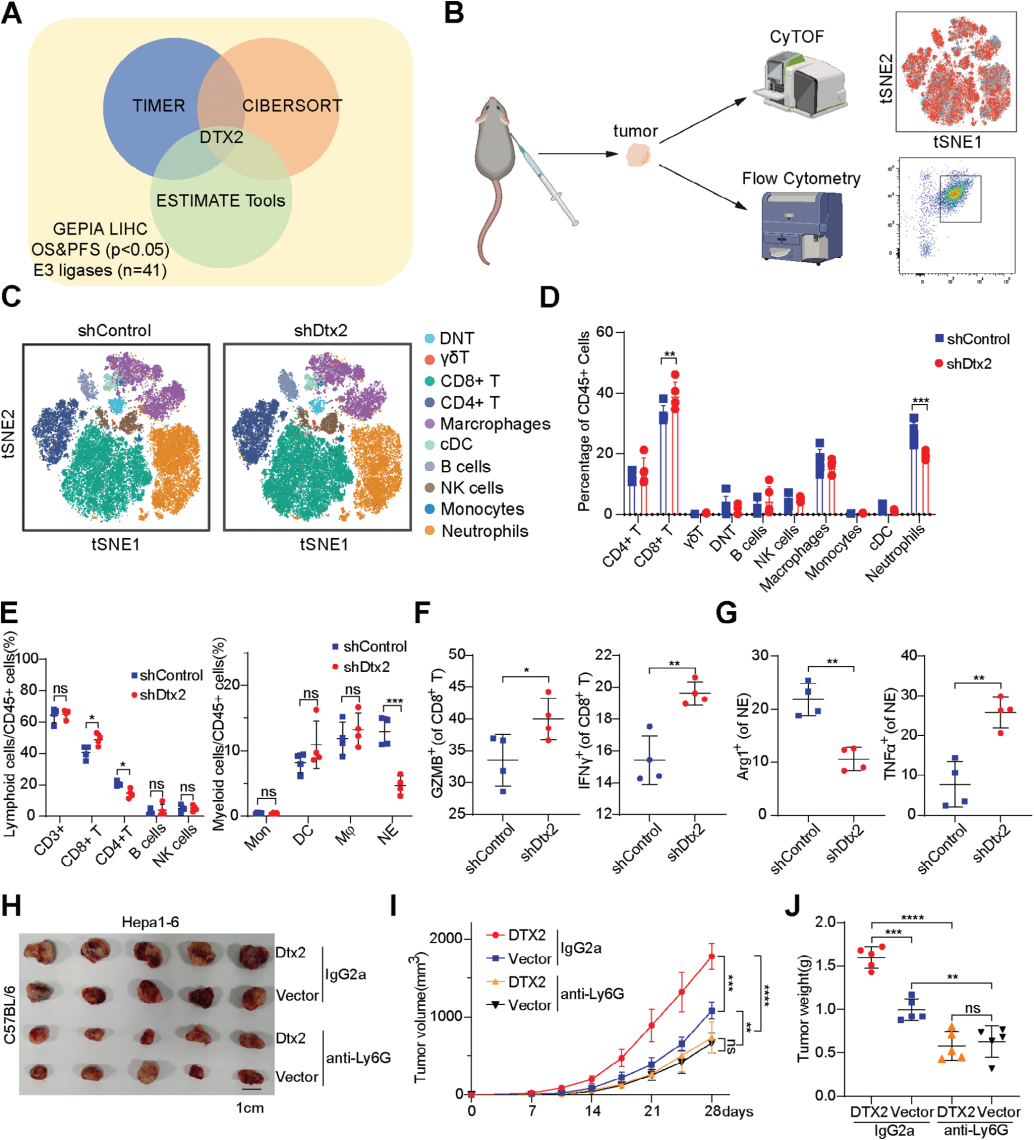
DTX2 in HCC cells promotes tumor progression by affecting TANs and CD8+ T cells. (A) Diagram of the screening process for DTX2. (B) Schematic diagram of mouse subcutaneous tumor analysis. (C) t‐SNE plot of infiltrating immune cell clusters. (D) Distribution of immune cells infiltrating subcutaneous tumors (n = 5 per group). (E) Distribution of immune cells by flow cytometry (*n* = 4 per group). (F) Proportions of GZMB+ CD8+ T cells and IFN+ CD8+ T cells among total CD8+ T cells. (G) Proportions of ARG1+ cells and TNFα+ cells among total neutrophils. (H‐J) Images (H), growth curves (I) and burdens (J) of subcutaneous tumors (n = 5 per group). The data are presented as the means ± SDs. ^*^ *p* < 0.05, ^**^ *p* < 0.01, ^***^ *p* < 0.001, ^****^ *p* < 0.0001. DNT, double‐negative T cell.

To identify ubiquitin ligases impacting immune cell infiltration in HCC tissues, we retrieved 445 reviewed genes encoding human ubiquitin ligases from the UniProt database. Through the GEPIA and TIMER web server, we identified 41 potential genes by comparing the expression of genes in tumor tissues with that in adjacent tissues and analyzing the overall survival and recurrence‐free survival (Extended file. 1). Subsequently, via TIMER and the CIBERSORT algorithm, DTX2, Cyclin‐F and were found to be highly correlated with immune cell infiltration in HCC tissues (Figure S1A,B, Supporting Information). To eliminate the effect of stromal cells in HCC tissue on immune cell infiltration, we further found that DTX2 had a weak correlation with the stromal cell score according to the ESTIMATE tool (Figure S1C, Supporting Information). Therefore, we hypothesized that DTX2 in HCC cells affects immune cell infiltration in HCC tissues (**Figure** **1**A; Figure S2A, Supporting Information). In addition, TIMER revealed high DTX2 expression in diverse tumors (Figure S2B, Supporting Information). Moreover, the difference in tumor growth rate between C57BL/6 mice and NOD/SCID mice suggested that DTX2 promoted tumor growth by influencing the immune microenvironment (Figure S3A–F, Supporting Information).

Subsequently, we constructed subcutaneous tumors and used them for flow cytometry analysis and cytometry by time of flight (CyTOF) detection (Figure 1B). CyTOF identified different immune cells in subcutaneous tumors of C57BL/6 mice (Figure 1C). Knockdown of Dtx2 in Hepa1‐6 cells led to an increase in the proportion of CD8+ T cells and a decrease in the proportion of neutrophils (Figure 1D). After dimensionality reduction via t‐distributed stochastic neighbor embedding (t‐SNE), we found that the expression of CD8a was increased but that of CD11b and Gr‐1 was decreased, consistent with the changes of corresponding immune cells (Figure S2C, Supporting Information). Immunohistochemical (IHC) staining showed consistent results (Figure S4A, Supporting Information). Besides, the expression of PD‐1 in CD8+ T cells was decreased in tumors generated from Hepa1‐6 shDtx2 cells, while the expression of Granzyme B (GZMB) and MHC‐II was increased (Figure S2D, Supporting Information). Another cluster analysis of the TANs revealed that the expression of Ki67 was slightly increased in the Hepa1‐6 shDtx2 group, but no significant differences in the other markers were detected (Figure S2E, Supporting Information). Flow cytometry (Figure S5A,B, Supporting Information) were used to confirm that knockdown of Dtx2 resulted in an increased proportion of CD8+ T cells and a decreased proportion of TANs (Figure 1E). In addition, Dtx2 knockdown increased the proportions of interferon‐γ (IFNγ)+ CD8+ T cells and GZMB+ CD8+ T cells within the overall population of CD8+ T cells (Figure 1F). Moreover, Dtx2 knockdown decreased the proportion of ARG1+ TANs and increased the proportion of tumor necrosis factor‐α (TNFα)+ TANs (Figure 1G). These results suggest that knockdown of Dtx2 in Hepa1‐6 cells increases the cytotoxicity of CD8+ T cells while inhibiting the polarization of TANs toward a protumor phenotype. Furthermore, depletion of mouse neutrophils with anti‐Ly6G weakened the protumor effect of Dtx2 overexpression (Figure 1H–J). IHC staining showed that the depletion of neutrophils blocked the effect of Dtx2 overexpression on CD8+ T cell infiltration (Figure S4B, Supporting Information). As changes in the proportion of CD4+ T cells were also detected by flow cytometry, we used anti‐CD8a and anti‐CD4 to deplete CD8+ T cells and CD4+ T cells, respectively (Figure S3G, Supporting Information). The results suggested that depletion of CD8+ T cells weakened the inhibitory effect of Dtx2 knockdown on tumor growth, while depletion of CD4+ T cells had no such effect (Figure S3H–J, Supporting Information). IHC staining showed that depletion of CD8+ T cells or CD4+ T cells did not affect TANs infiltration, suggesting that TANs affect CD8+ T cell infiltration into tumors (Figure S4C, Supporting Information).

Whether DTX2 can directly promote the growth of HCC cells remained unclear. Therefore, we constructed human and mouse HCC cell lines with stable knockdown or overexpression of DTX2 via lentiviral transduction (Figure S6A–D, Supporting Information). Colony formation and CCK‐8 proliferation assays showed that modulation of DTX2 expression in HCC cells did not significantly affect the growth of tumor cells (Figure S6E–H, Supporting Information). Similar results were obtained in the EdU incorporation assay (Figure S7A,B, Supporting Information).

### 2.2 DTX2 Weakens the Proliferation and Cytotoxicity of CD8+ T Cells by Increasing Neutrophil Chemotaxis and Polarization Toward the Protumor Phenotype

**Figure 2:**
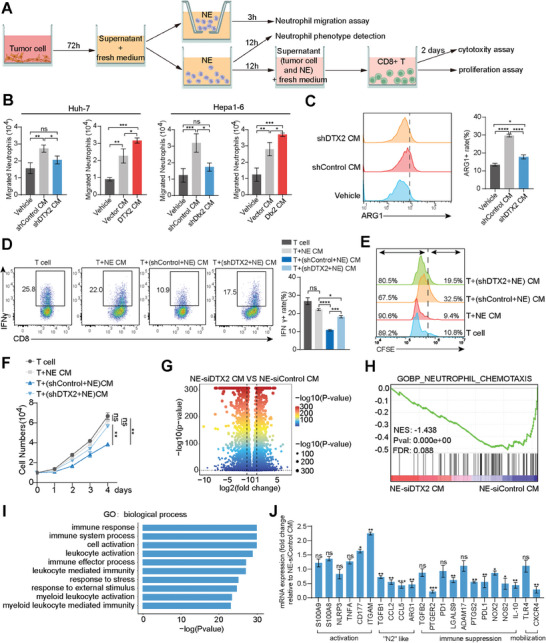
DTX2 attenuates CD8+ T cells by increasing neutrophil chemotaxis and protumoral polarization. (A) Schematic diagram of the in vitro biological functional assays. (B) Neutrophil migration assays using indicated groups of medium. (C) ARG1 expression level in neutrophils measured by flow cytometry. The dotted line indicates the boundary between ARG1‐ and ARG1+ cells. (D) IFNγ expression in CD8+ T cells measured by flow cytometry. (E) CFSE staining of CD8+ T cells. The dotted line indicates the CFSE staining peak in nonproliferating cells. (F) The cell counting number of CD8+ T cells. (G) Volcano plot of RNA‐seq data from neutrophils cultured with Huh‐7 siControl cell CM or Huh‐7 siDTX2 cell CM. (H) GSEA of neutrophil chemotaxis‐related gene sets in RNA‐seq data. (I) Biological process analysis of differentially expressed genes in RNA‐seq. (J) Phenotypic markers of neutrophils cultured with Huh‐7 siControl cell CM or Huh‐7 siDTX2 cell CM were analyzed by qPCR. The data are presented as the means ± SDs. ^*^ *p* < 0.05, ^**^ *p* < 0.01, ^***^ *p* < 0.001, ^****^ *p* < 0.0001. NE, neutrophil; GOBP, Gene Ontology biological process; ns, nonsignificant difference.

Related functional experiments were performed on neutrophils and CD8+ T cells in vitro (**Figure** **2**A). The neutrophil migration assay showed that the culture supernatant of HCC cells with DTX2 knockdown weakened neutrophil chemotaxis without affecting neutrophil apoptosis (Figure 2B; Figure S8A, Supporting Information). The qPCR detection of showed that ARG1 and transforming growth factor‐β (TGF‐β) were significantly increased in migrated neutrophils attracted by culture medium of tumor cell overexpressing DTX2, which indicated the pro‐tumoral characteristic of these migrated neutrophils (Figure S8B, Supporting Information). In addition, the culture supernatant of HCC cells with DTX2 knockdown inhibited ARG1 expression in neutrophils (Figure 2C). We found that the expression level of IFNγ in CD8+ T cells was partially restored when the cells were cultured in the culture supernatant of DTX2‐knockdown HCC cells and neutrophils (Figure 2D). However, there was no significant difference in the IFNγ expression level between CD8+ T cells cultured in culture medium from wild‐type tumor cells and those cultured in culture medium from DTX2‐knockdown cells (Figure S8C, Supporting Information). CFSE staining showed that the culture supernatant from wild‐type tumor cells and neutrophils inhibited the proliferation of CD8+ T cells, while culture supernatant from DTX2‐knockdown tumor cells and neutrophils partially restored the proliferation of CD8+ T cells (Figure 2E). Similar results were obtained by counting CD8+ T cells (Figure 2F), and no significant difference was found in the proliferation of CD8+ T cells cultured in medium from wild‐type tumor cells and those cultured in medium from DTX2‐knockdown cells (Figure S8D, Supporting Information). This finding suggests that the effect of DTX2 in HCC cells on CD8+ T cells is dependent on neutrophils as mediators.

We performed RNA‐seq analysis on neutrophils (Figure 2G) and found that genes related to neutrophil chemotaxis were largely downregulated in neutrophils cultured in the supernatant of HCC cells with DTX2 knockdown (Figure 2H). Biological process enrichment analysis showed that the differentially expressed genes exhibited enrichment mainly in the terms of immune response and myeloid leukocyte activation (Figure 2I). According to published literature on neutrophil function, neutrophils cultured in the supernatant of HCC cells with DTX2 knockdown exhibited increased transcript levels of genes related to innate immune activation.^[^ ^12^, ^13^, ^14^ ^]^ However, genes related to the protumor phenotype of neutrophils (“N2”‐like genes) or immunosuppression and cell mobilization were downregulated (Figure 2J).^[^ ^1^, ^15^, ^16^ ^]^ Flow‐sorted TANs in subcutaneous tumors revealed similar results (Figure S8E, Supporting Information). These findings suggest that DTX2 increases chemotaxis and protumor polarization of neutrophils.

### 2.3 Targeting CXCL2 and CXCL6 in Tumor Cells or CXCR1 and CXCR2 in Neutrophils Partially Blocks the Effect of DTX2 in HCC Cells on Immune Cells

**Figure 3:**
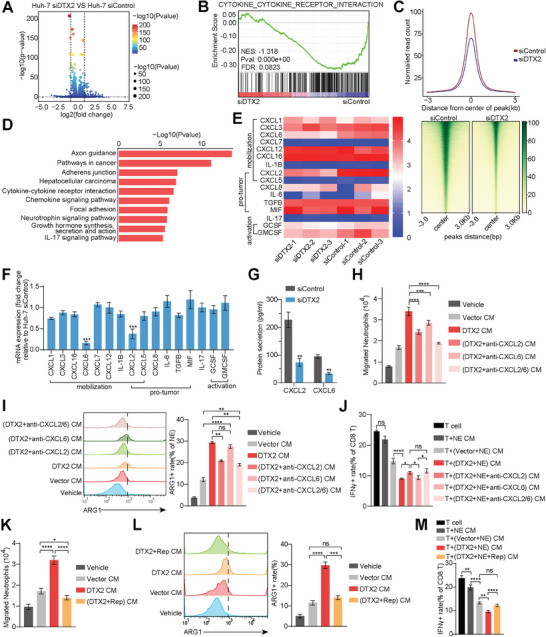
Targeting CXCL2/CXCL6‐CXCR1/CXCR2 axis reduces the effects of DTX2 on neutrophils and CD8+ T cells. (A) Volcano plot of RNA‐seq data from the Huh‐7 siControl and Huh‐7 siDTX2 groups. (B) GSEA of the cytokine‐cytokine receptor interaction gene set in RNA‐seq data. (C) Peak signal distribution diagram and peak distribution heatmap based on ATAC‐seq of the Huh‐7 siControl group and the Huh‐7 siDTX2 group. (D) KEGG pathway analysis of the 116 downregulated genes. (E) Heatmaps of neutrophil‐related cytokines and chemokines in RNA‐seq data. (F) The expression levels of neutrophil‐related cytokines and chemokines measured by qPCR. (G) The concentrations of secreted CXCL2 and CXCL6 measured by ELISA. (H) Neutrophil migration assays using indicated groups of medium. (I) ARG1 expression level in neutrophils measured by flow cytometry. The dotted line indicates the boundary between ARG1‐ and ARG1+ cells. (J) IFNγ expression in CD8+ T cells measured by flow cytometry. (K) Neutrophil migration assays using indicated groups of medium. (L) ARG1 expression in neutrophils measured by flow cytometry. The dotted line indicates the boundary between ARG1‐ and ARG1+ cells. (M) IFNγ expression level in CD8+ T cells in each group measured by flow cytometry. The data are presented as the means ± SDs. ^*^ *p* < 0.05, ^**^ *p* < 0.01, ^***^ *p* < 0.001, ^****^ *p* < 0.0001. Rep, Reparixin; ns, nonsignificant difference.

A total of 189 downregulated genes and 117 upregulated genes were detected from RNA‐seq analysis in Huh‐7 cells with DTX2 knockdown (fold change > 2, P < 0.05) (**Figure** **3**A). Gene Set Enrichment Analysis (GSEA) showed that genes related to cytokine‐cytokine receptor interactions in the DTX2 knockdown group were downregulated (Figure 3B). Assay for Transposase Accessible Chromatin with high throughput sequencing (ATAC‐seq) Granulocyte‐Macrophage Colony‐Stimulating Factor revealed that the chromatin accessibility in Huh‐7 cells with DTX2 knockdown was decreased (Figure 3C). Subsequently, we identified 116 genes with reduced chromatin accessibility and decreased transcript levels. Kyoto Encyclopedia of Genes and Genomes (KEGG) enrichment analysis revealed that this gene set exhibited enrichment in the cytokine‐cytokine receptor interaction pathway and chemokine signaling pathway (Figure 3D). Considering the above results, we focused on chemokines and cytokines that affect neutrophils.^[^ ^17^, ^18^ ^]^ RNA‐seq showed that the transcript levels of CXCL2, CXCL3, and CXCL6 were decreased in DTX2 knockdown group (Figure 3E). qPCR and enzyme‐linked immunosorbent assay (ELISA) further demonstrated that knockdown of DTX2 exhibited the greatest inhibitory effects on the transcription and secretion of CXCL2 and CXCL6 (Figure 3F,G). Knockdown of Dtx2 in Hepa1‐6 cells resulted in the same effects (Figure S9A,B, Supporting Information).

We used anti‐CXCL2 and anti‐CXCL6 to neutralize the corresponding chemokines in the culture medium of tumor cells. The neutrophil migration assay showed that the ability of DTX2 overexpression in HCC cells to promote neutrophil chemotaxis was partially blocked after neutralization of CXCL2 or CXCL6 without affecting the apoptosis of neutrophils (Figure 3H; Figure S9C, Supporting Information). The influence of simultaneous neutralization of these two chemokines was more obvious than that of neutralization of either alone (Figure 3H). Neutralization of CXCL2 partially weakened the promoting effect of DTX2 overexpression in HCC cells on ARG1 expression in neutrophils, while neutralization of CXCL6 had no such effect (Figure 3I). Neutralizing CXCL2 in the culture supernatant of DTX2‐overexpressing tumor cells and neutrophils partially restored IFNγ expression and proliferation of CD8+ T cells, while neutralizing CXCL6 did not (Figure 3J; Figure S9D, Supporting Information). Similarly, neutralizing mouse CXCL2 and mouse CXCL6 had the same effects (Figure S9G–I, Supporting Information).

Since the corresponding receptors for CXCL2 and CXCL6 on neutrophils are CXCR1 and CXCR2, we used reparixin, a CXCR1/CXCR2 receptor antagonist, for further research. Reparixin effectively blocked the increase in neutrophil chemotaxis induced by DTX2 overexpression in HCC cells (Figure 3K) without affecting neutrophil apoptosis (Figure S9E, Supporting Information). Reparixin partially blocked the overexpression of ARG1 in neutrophils (Figure 3L). It also showed promising effects on promoting the cytotoxicity and proliferation of CD8+ T cells (Figure 3M; Figure S9F, Supporting Information). The addition of reparixin to the medium of Hepa1‐6 cells also had the same effects (Figure S9J–K, Supporting Information). The acceleration of tumor growth was abolished after treatment with reparixin (Figure S9L,M, Supporting Information). Multiplex immunofluorescence staining of tumor sections from the Dtx2‐overexpressing group not treated with reparixin revealed that CD8+ T cells, especially IFNγ+ CD8+ T cells, were located mainly at the tumor margins (white arrow point). The CD8+ T cells was excluded from the neutrophil‐infiltrated area, as indicated by Ly6G staining (orange arrow point). After treated with reparixin, subcutaneous tumor contained fewer Ly6G+ cells and more CD8+ IFN+ cells (white arrow point, Figure S9N, Supporting Information).

### 2.4 DTX2 Regulates the Transcription of CXCL2 and CXCL6 by Affecting H2BK120ub1

**Figure 4:**
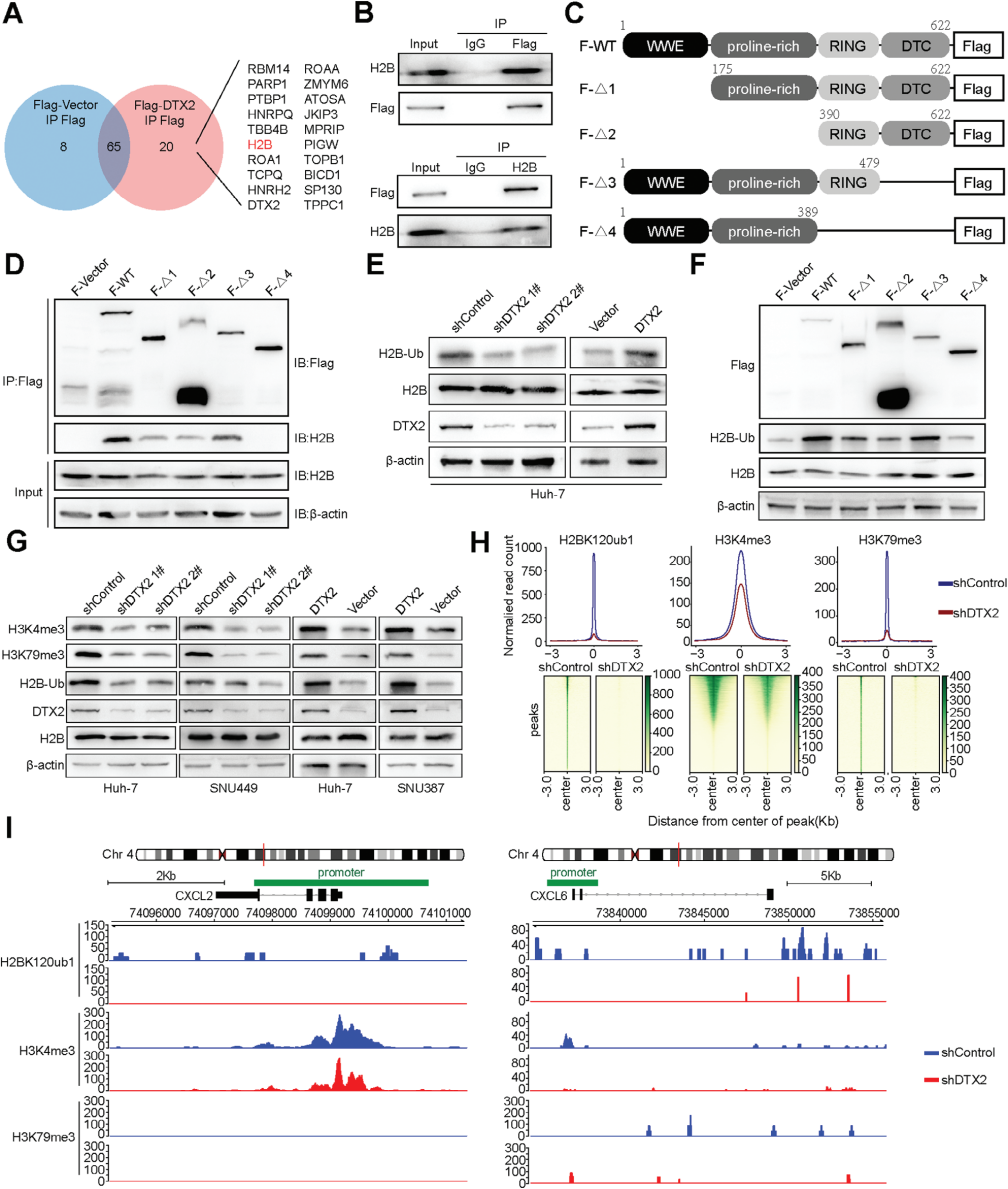
DTX2 affects H2B‐Ub, H3K4me3, and H3K79me3 and regulates the transcription of CXCL2 and CXCL6. (A) Protein profile obtained via mass spectrometry of different groups of co‐IP. (B) Co‐IP followed by western blot with anti‐FLAG or anti‐H2B. (C) Schematic diagram of the construction of the human DTX2 truncation mutant plasmids. (D) Exogenous expression of the truncated DTX2 proteins in Huh‐7 cells followed by co‐IP with anti‐FLAG. (E) Western blot analysis of H2B‐Ub. (F) Western blot analysis of H2B‐Ub in different groups of Huh‐7 cells expressing the truncated DTX2 proteins. (G) Western blot analysis of H2B‐Ub, H3K4me3 and H3K79me3. (H) Peak signal distribution diagram and peak distribution heatmap based on the CUT&Tag results obtained using anti‐H2B‐Ub, anti‐H3K4me3 and anti‐H3K79me3 in the Huh‐7 shControl group and the Huh‐7 shDTX2 group. (I) IGV diagram based on the CUT&Tag results obtained using anti‐H2B‐Ub, anti‐H3K4me3 and anti‐H3K79me3 at the CXCL2 and CXCL6 genomic loci. Chr, chromosome.

The co‐immunoprecipitation assay and mass spectrometry analysis revealed 20 potential proteins that specifically interact with DTX2. Considering the finding that DTX2 can affect chromatin accessibility in HCC cells, histone H2B was selected for verification (**Figure** **4**A). Western blot suggested that histone H2B interacted with DTX2 (Figure 4B). We constructed plasmids expressing DTX2 truncation mutants (Figure 4C) and found that the loss of the RING domain of DTX2 resulted in the inability of DTX2 to interact with histone H2B (Figure 4D). It has been reported that histone H2B can be degraded via polyubiquitination at lysine 120.^[^ ^19^ ^]^ Western blot verified that DTX2 did not affect various types of H2B polyubiquitination (Figure S10A,B, Supporting Information). Subsequently, we found that DTX2 affected monoubiquitination of histone H2B at lysine 120 (H2BK120ub1, H2B‐Ub) (Figure 4E). The absence of the RING domain significantly weakened the effect of DTX2 on H2BK120ub1 (Figure 4F).

Various studies have reported that H2BK120ub1 is closely related to the polymethylation of histone H3 at lysines 4 and 79, both of which could be epigenetic markers for quantification of transcriptional activity.^[^ ^6^ ^]^ We verified that DTX2 promoted H2BK120ub1, H3K4me3, and H3K79me3 (Figure 4G). CUT&Tag showed that knockdown of DTX2 significantly decreased the distribution intensity of H2BK120ub1, H3K4me3 and H3K79me3 signals across the genome (Figure S10C, Supporting Information). Similarly, knockdown of DTX2 significantly decreased the distribution of H2BK120ub1, H3K4me3, and H3K79me3 peaks across the genome (Figure 4H). Peak distribution indicated that H3K4me3 was clearly enriched in gene promoter functional regions (Figure S10D, Supporting Information). Integrative Genomics Viewer (IGV) revealed that the distribution of H2BK120ub1 and H3K4me3 signals within the CXCL2 and CXCL6 genomic loci were significantly reduced in DTX2‐knockdown cells, with the H3K4me3 signals concentrated in the promoter regions of CXCL2 and CXCL6 (Figure 4I). This result was more clearly demonstrated in the triple replication experiments of CUT&Tag for H3K4me3 (Figure S10E, Supporting Information). The results of chromatin immunoprecipitation followed by qPCR (ChIP‐qPCR) verified that the binding ratios of H2BK120ub1 and H3K4me3 to gene fragments in the regions with high read distributions were reduced in the DTX2 knockdown group (Figure S10F, Supporting Information).

### 2.5 DTX2 is Overexpressed in HCC and is Associated with Poor Prognosis

**Figure 5:**
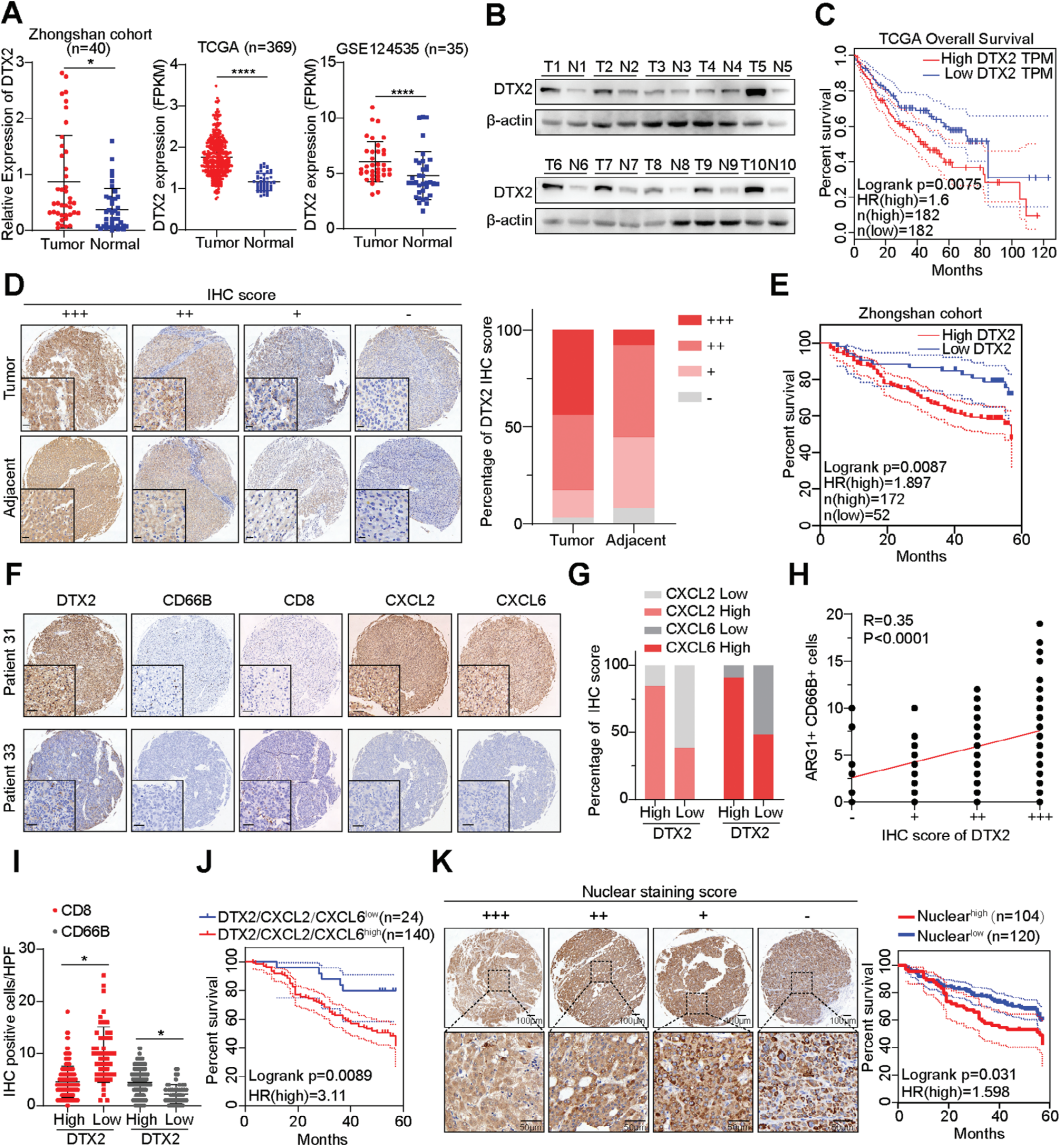
DTX2 is overexpressed in HCC and indicates poor prognosis. (A) The mRNA level of DTX2 from Zhongshan cohort, the expression level (FPKM) of DTX2 from TCGA LIHC dataset and GSE124535 dataset. (B) The DTX2 protein levels from Zhongshan cohort. (C) Overall survival curves of HCC patients in TCGA. (D) Representative diagram and statistical graph of the DTX2 staining intensity in the tissue microarray from Zhongshan cohort (*n* = 224 samples). (E) Overall survival curves of HCC patients from Zhongshan cohort (corresponding to DTX2 staining in the tissue microarray). (F) Images of IHC staining for DTX2, CD66B, CD8, CXCL2 and CXCL6 in the HCC tissue microarray samples from patient No. 31 and patient No. 33. (G) Statistical analysis of the intensity and proportion of CXCL2 and CXCL6 staining in Zhongshan cohort (corresponding to DTX2 staining in the tissue microarray, *n* = 224 samples). (H) Correlation analysis between IHC score of DTX2 and the number of ARG1+ CD66B+ cells on HCC tissue microarray after multiplex immunofluorescence staining. (I) Statistical graph of the number of CD8‐ and CD66B‐positive cells in different groups (corresponding to DTX2 staining in the tissue microarray, *n* = 224 samples). (J) Overall survival curves of HCC patients at Zhongshan Hospital divided into the indicated groups (corresponding to IHC staining in the tissue microarray). (K) Representative images of the DTX2 nuclear staining intensity in the tissue microarray. Overall survival curves of HCC patients at Zhongshan Hospital divided into the indicated groups (corresponding to IHC staining in the tissue microarray). The data are presented as the means ± SDs. ^*^ *p* < 0.05, ^***^ *p* < 0.001, ^****^ *p* < 0.0001.

DTX2 was overexpressed in HCC tissues in Zhongshan cohort. In addition, analysis of RNA‐seq from both the TCGA‐LIHC cohort and GSE124535 dataset showed that DTX2 was overexpressed in HCC tissues (**Figure** **5**A). We then randomly selected 10 pairs of HCC and adjacent liver tissues and found that DTX2 was highly expressed in most of the HCC tissues (Figure 5B). TCGA‐LIHC data revealed that high DTX2 expression in HCC tissues indicated worse overall survival (Figure 5C). IHC staining of HCC tissue microarray in Zhongshan cohort revealed that a large percentage of the tumor tissues (≈75%) exhibited high DTX2 expression (++/+++) (Figure 5D). Correspondingly, patients with high DTX2 expression had worse overall survival (Figure 5E). Moreover, HCC tissues with high DTX2 expression had greater infiltration of TANs, stronger staining of CXCL2 and CXCL6, and less infiltration of CD8+ T cells (Figure 5F). In addition, high expression of CXCL2 or CXCL6 was more common in HCC patients with high DTX2 expression (Figure 5G). ARG1+ CD66B+ cells of each tissue were counted on HCC tissue microarray after multiplex immunofluorescence staining. Correlation analysis showed that IHC score of DTX2 was positively correlated with the number of ARG1+ CD66B+ cells in tumor tissues (Figure 5H). The number of infiltrated CD8+ T cells was lower in HCC tissues with higher DTX2 expression, while the number of TANs in these tissues was greater (Figure 5I). We used IHC staining data for DTX2, CXCL2, and CXCL6 to group HCC patients and found that patients with high expression of DTX2, CXCL2, and CXCL6 had worse overall survival (Figure 5J). IHC staining of the tissue microarray also revealed differences in the distribution of DTX2 in the nucleus of HCC cells. High DTX2 staining in the nucleus of HCC cells indicated worse overall survival (Figure 5K). Subsequently, we used univariate and multivariate logistic regression models to analyze the relationships between clinical parameters and DTX2 expression in HCC patients. Tumor size, the expression level of DTX2, and vascular invasion had statistically significant effects on the overall survival of HCC patients (Figure S11A,B, Supporting Information). Univariate and multivariate Cox regression analyses suggested that missing of tumor capsule, a large tumor size, and a high DTX2 expression level could be independent risk factors used to predict survival time in patients (Figure S11C,D, Supporting Information).

### 2.6 Inhibitor of Mouse DTX2 Suppresses Cxcl2 and Cxcl6 Transcription and Tumor Growth

**Figure 6:**
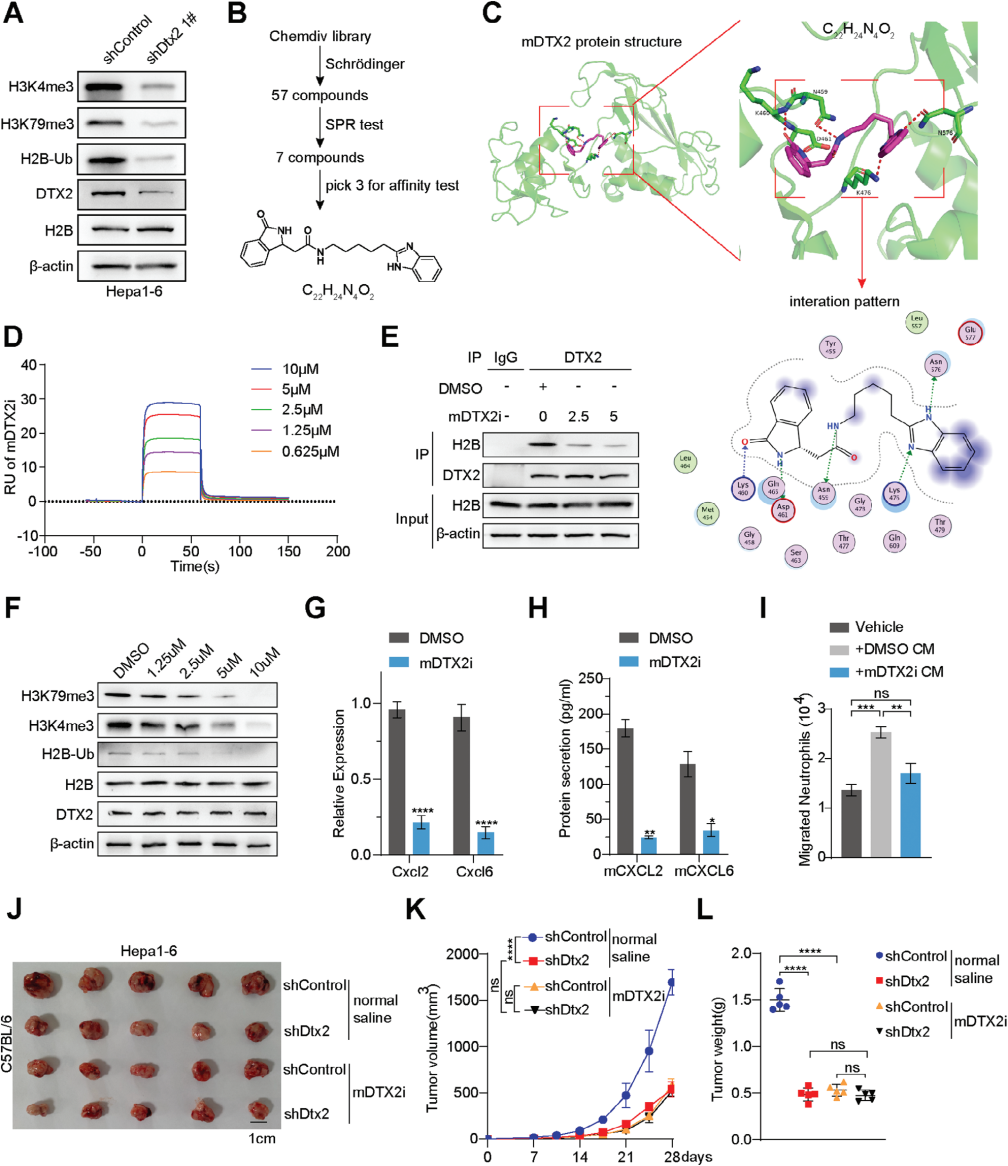
Small molecule compound targeting mouse DTX2 (mDTX2) inhibits Cxcl2 and Cxcl6 transcription and tumor growth. (A) Western blot analysis of H2B‐Ub, H3K4me3, and H3K79me3. (B) Flow chart of the screen for small molecule compounds targeting mDTX2. (C) Schematic diagram of the predicted docking structure of C_22_H_24_N_4_O_2_ and mDTX2. (D) SPR analysis of the mDTX2i. (E) Co‐IP of Hepa1‐6 cells with anti‐DTX2. (F) Western blot analysis of H2B‐Ub, H3K4me3, and H3K79me3 in Hepa1‐6 cells. (G) The transcript levels of Cxcl2 and Cxcl6 in Hepa1‐6 cells treated with DMSO or the mDTX2i (5 µm) measured by qPCR. (H) The secretion of mouse CXCL2 and mouse CXCL6 from Hepa1‐6 cells treated with DMSO or the mDTX2i (5 µm) measured by ELISA. (I) Neutrophil chemotaxis in the Hepa1‐6 DMSO CM and Hepa1‐6 mDTX2i CM groups. (J–L) Images (J), growth curve (K) and burden (L) of subcutaneous tumors formed from Hepa1‐6 shControl and Hepa1‐6 shDtx2 cells treated with normal saline or the mDTX2i (*n* = 5 per group). The data are presented as the means ± SDs. ^*^ *p* < 0.05, ^**^ *p* < 0.01, ^***^ *p* < 0.001, ^****^ *p* < 0.0001. C_22_H_24_N_4_O_2_, *N‐[5‐(1H‐benzimidazol‐2‐YL) pentyl]‐2‐(3‐oxo‐2,3‐dihydro‐1H‐isoindol‐1‐yl) acetamide*; mDTX2i, mouse DTX2 inhibitor; ns, nonsignificant difference.

In Hepa1‐6 cells, Dtx2 knockdown also reduced the abundances of H2BK120ub1, H3K4me3, and H3K79me3 (**Figure** **6**A). Then, we screened small molecule compounds to identify effective inhibitors targeting the RING domain of mouse DTX2. We identified the 454–470 in the RING domain for virtual screening via Schrödinger, and 175 compounds were selected. Among 175 compounds, the top 60 according to the predicted binding affinity were selected for procurement, and 57 of these were obtained. Then surface plasmon resonance (SPR) experiments were performed and 7 compounds with signal intensities (RU) > 20 were selected (Figure S12A, Supporting Information). The three compounds with the highest signal intensities were selected for evaluation of their binding affinity for DTX2. Among these compounds, *N‐[5‐(1H‐benzimidazole‐2‐yl) pentyl]‐2,3‐dihydro‐3‐oxo‐1H‐isoindole‐1‐acetamide* (ChemDiv Num: Y043‐4427; C_22_H_24_N_4_O_2_) had the smallest equilibrium dissociation constant (K_D_) and the largest association rate constant (Ka), indicating the highest affinity for DTX2 (Figure S12B, Supporting Information). Two compounds (ChemDiv Num: Y043‐4427 and Y501‐5662) were found to inhibit tumor growth (Figure S12C,D, Supporting Information). Based on the above results, C_22_H_24_N_4_O_2_ was selected as the mouse DTX2 inhibitor (mDTX2i) (Figure 6B).

C_22_H_24_N_4_O_2_ was found to bind to the mouse DTX2 protein and form hydrogen bonds at asparagine 459, lysine 460, aspartate 461, lysine 476, and asparagine 576 (Figure 6C). SPR analysis revealed that C_22_H_24_N_4_O_2_ had a high affinity for the mouse DTX2 protein at concentrations ranging from 5–10 µm (Figure 6D). The half‐maximal inhibitory concentration (IC50) in Hepa1‐6 cells was 30.83 µm (Figure S12E, Supporting Information). Co‐IP revealed that the binding of DTX2 to histone H2B decreased gradually after the addition of increasing concentrations of the mDTX2i (Figure 6E), accompanied by decreased abundances of H2BK120ub1, H3K4me3, and H3K79me3 (Figure 6F). The mDTX2i treatment inhibited the transcription and secretion of mouse CXCL2 and mouse CXCL6 (Figure 6G,H). In addition, mDTX2i treatment on tumor cells inhibited neutrophil chemotaxis and ARG1 expression of neutrophils (Figure 6I; Figure S12F, Supporting Information). Furthermore, the addition of mDTX2i increased the cytotoxicity of CD8+ T cells (Figure S12G, Supporting Information). Similarly, mDTX2i treatment did not change the polyubiquitination of histone H2B (Figure S12H, Supporting Information). Subcutaneous tumor model demonstrated that treatment with the mDTX2i did not further inhibit tumor growth in the Hepa1‐6 shDtx2 group (Figure 6J–L). IHC staining revealed that treatment with mDTX2i increased the infiltration of CD8+ T cells, decreased the infiltration of TANs, and decreased the expression of CXCL2 and CXCL6 in subcutaneous tumors (Figure S12I, Supporting Information).

### 2.7 Targeting DTX2 Sensitizes HCC Cells to PD‐1 Antibody

**Figure 7:**
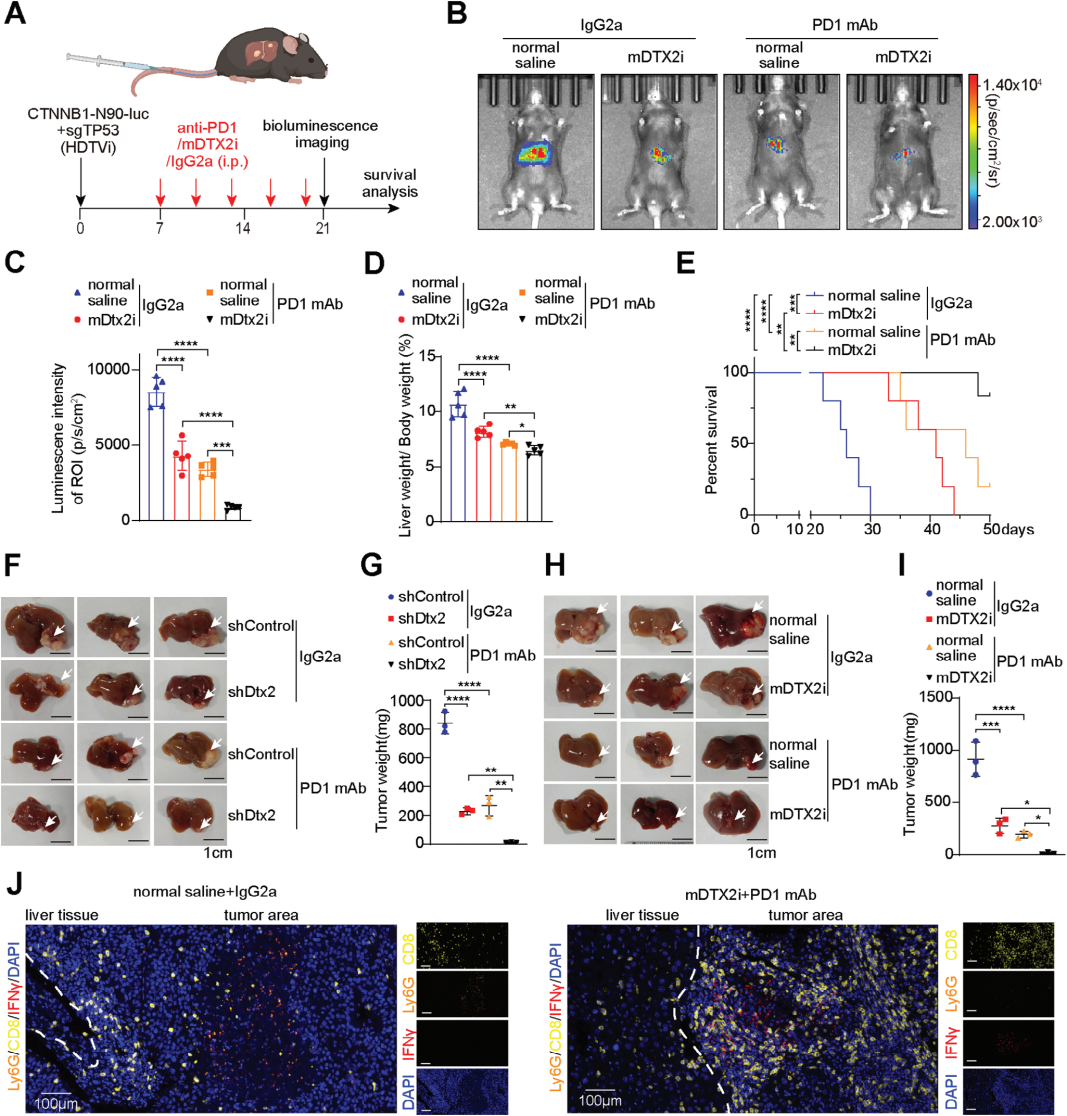
mDTX2i treatment sensitizes HCC cells to PD1 antibody treatment. (A) Schematic diagram of establishing the spontaneous HCC model with CTNNB1‐N90‐luc/sgTP53 plasmids via HDTVi. (B) Representative in vivo bioluminescence images of mice. (C) Luminescence intensity of the ROIs (*n* = 5 per group). (D) Liver weight/body weight ratio in the indicated groups (*n* = 5 per group). (E) Survival curves of the mice in the indicated groups (*n* = 5 per group). (F,G) Images (F) and burden (G) of orthotopic liver tumors formed from Hepa1‐6 shControl and Hepa1‐6 shDtx2 cells treated with IgG2a or an anti‐PD1 (*n* = 3 per group). (H,I) Images (H) and burden (I) of orthotopic liver tumors formed from Hepa1‐6 cells treated with IgG2a, the anti‐PD1, saline, or the mDTX2i (*n* = 3 per group). (J) Multiplex immunofluorescence staining of Ly6G, CD8, IFNγ and DAPI in indicated groups of orthotopic liver tumors. The data are presented as the means ± SDs. ^*^ *p* < 0.05, ^**^ *p* < 0.01, ^***^ *p* < 0.001, ^****^ *p* < 0.0001. HDTVi, hydrodynamic tail vein injection; mAb, mouse antibody; mDTX2i, mouse DTX2 inhibitor; ns, nonsignificant difference.

We further explored whether mDTX2i treatment can increase the efficacy of ICIs. To this end, we established a model of spontaneous HCC model based on methods described in published literature^[^ ^20^, ^21^ ^]^ (**Figure** **7**A). Bioluminescence imaging demonstrated that the mDTX2i combined with PD‐1 antibody significantly inhibited the growth of spontaneous tumors (Figure 7B,C). This combination therapy decreased the tumor burden and prolonged the survival of treated mice (Figure 7D,E). Furthermore, the results in the subcutaneous tumor model showed that tumor growth was inhibited more significantly in the Hepa1‐6 shDtx2 group than in the Hepa1‐6 wild‐type group following treatment with the PD‐1 antibody, and this effect was accompanied by a lower tumor burden and longer overall survival time (Figure S13A–D, Supporting Information). Similarly, mice treated with the combination of the mDTX2i and the PD‐1 antibody had smaller tumors and longer survival times than those treated with PD‐1 monotherapy (Figure S13E–H, Supporting Information). IHC staining revealed that the infiltration of CD8+ T cells and the number of IFNγ‐positive cells were obviously increased in the group treated with the mDTX2i and PD‐1 antibody combination (Figure S13I, Supporting Information). In the orthotopic tumor model, knockdown of Dtx2 or treatment with the mDTX2i increased the efficacy of the PD‐1 antibody (Figure 7F–I). Multiplex immunofluorescence staining of the orthotopic tumors revealed that combination treatment with the mDTX2i and the PD‐1 antibody increased the infiltration of CD8+ IFNγ+ cells. Almost no TANs (Ly6G+ cells) were observed in these orthotopic tumors (Figure 7J; Figure S13J, Supporting Information). Besides, the combined therapy or monotherapy showed satisfied biosafety in orthotopic tumor model (Figure S13K, Supporting Information).

Furthermore, flow cytometry also found the decreased expression of ARG1 in mouse neutrophils cultured in cell medium added with mDXT2i in vitro (Figure S14A, Supporting Information). Then qPCR showed that the transcription levels of Cxcl2 and Cxcl6 was also decreased in the group of neutrophils treated with mDXT2i (Figure S14B, Supporting Information). In orthotopic tumors, we did not find that the combined therapy could further significantly reduce the expression level of ARG1, CXCL2, and CXCL6 in neutrophils. However, the combined therapy enhanced the expression of IFNγ in CD8+ T cells, which is consistent with the result shown in Figure S13i (Supporting Information). This also suggests that mDTX2i improves the immune microenvironment by inhibiting the N2 phenotype of neutrophils.

## 3 Discussion

Recently, many E3 ligases have been found to target immune checkpoint proteins such as PD1, PD‐L1 and CTLA‐4 to disrupt the immune escape of tumor cells.^[^ ^22^, ^23^, ^24^ ^]^ Moreover, researchers have shown that some E3 ligases can inhibit the activities immune cells and promote tumor growth.^[^ ^7^, ^25^ ^]^ However, few studies have directly investigated the family of E3 ligases and their influence on the TME.

In this study, we focused on E3 ligases in HCC cells and verified that high expression of DTX2 in HCC cells promoted the infiltration of TANs and the polarization of neutrophils toward a protumor phenotype, accompanied by decreases in the infiltration and cytotoxicity of CD8+ T cells. Knockdown of DTX2 in HCC cells partially inhibited the chemotaxis and protumor polarization of neutrophils in vitro. Knockdown of DTX2 in HCC cells resulted in a decrease in the transcript level of PD‐L1, which was identified as a marker for immunosuppressive neutrophils in a previous study.^[^ ^26^ ^]^ This finding suggested that targeting DTX2 might attenuate the immunosuppressive characteristics of the immune microenvironment.

In addition to TGF‐β, Granulocyte‐Macrophage Colony‐Stimulating Factor (GM‐CSF), Interleukin‐6 (IL‐6), prostaglandin E2 (PGE2), hyaluronan fragments and hypoxia drive the polarization and accumulation of TAN2 neutrophils in HCC.^[^ ^27^, ^28^, ^29^ ^]^ In addition, most C‐X‐C chemokines produced by tumor cells and other cells in the TME possess a strong ability to recruit neutrophils into tumor tissues.^[^ ^30^ ^]^ In this study, we observed that knockdown of DTX2 led to the inhibition of CXCL2 and CXCL6 in HCC cells. However, we also found slight decreases in the transcription of TGF‐β, CXCL1, CXCL5 and IL‐1β via qPCR. Therefore, neutralizing CXCL2 and CXCL6 did not completely block the effects of DTX2 overexpression on neutrophils. In contrast, reparixin significantly attenuated neutrophil chemotaxis and polarization toward a protumor phenotype due to its broad‐spectrum antagonistic effects on CXCR1 and CXCR2.

Previous studies have demonstrated that chromatin accessibility is a major factor affecting gene transcription.^[^ ^31^, ^32^ ^]^ Histone epigenetic modifications can affect tumor progression by regulating chromatin accessibility.^[^ ^33^, ^34^ ^]^ We found that knockdown of DTX2 decreased chromatin accessibility in Huh‐7 cells. Subsequently, we discovered that DTX2 interacted with histone H2B and modulated H2BK120ub1 via its RING domain. Based on the crosstalk between H2BK120ub1 and H3 polymethylation, we proposed a mechanism explaining the increased transcription of CXCL2 and CXCL6 at the histone epigenetic modification level.

Substantial evidence indicates that TANs with a protumor phenotype attenuate the proliferation and cytotoxicity of effector T cells and contribute to resistance to ICIs.^[^ ^35^, ^36^ ^]^ Therefore, targeting DTX2 might increase the efficacy of anti‐PD‐1 antibody therapy in HCC. By screening, we identified a specific small molecule compound targeting the RING domain of mouse DTX2 as a DTX2 inhibitor. Further in vivo experiments confirmed that compared with anti‐PD1 monotherapy, combined treatment with mDTX2i and an anti‐PD1 antibody had better tumoricidal effects and prolonged the survival of mice without obvious side effects. Due to the differences in the amino acid sequences of the RING domain of mouse DTX2 and the RING domain of human DTX2, this inhibitor has not yet been proven suitable for use against human cells. However, this study provides a basis for the subsequent development of human DTX2 inhibitors.

**Figure 8:**
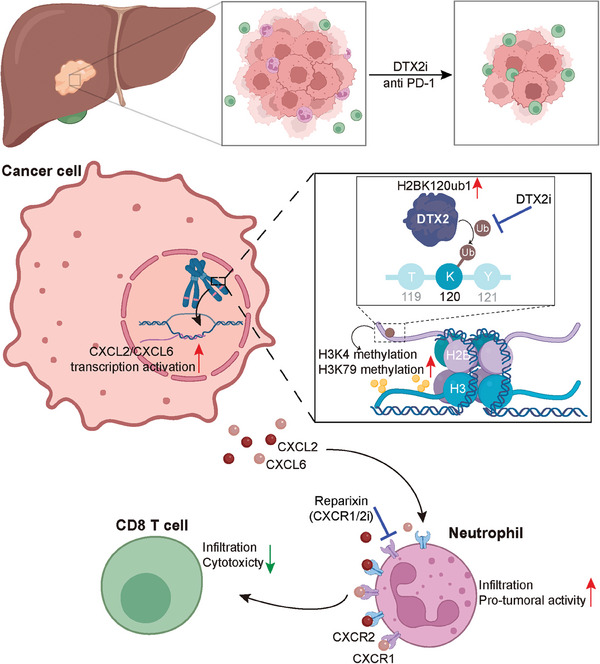
Mechanistic schematic diagram. DTX2 in HCC cells promotes H2BK120ub1, which in turn alters chromatin accessibility and promotes the transcription and secretion of CXCL2 and CXCL6. DTX2 promotes the recruitment of TANs and the polarization of neutrophils toward a protumor phenotype in tumor tissues, thereby inhibiting the tumoricidal effect of CD8+ T cells. Targeting DTX2 can attenuate the immunosuppressive characteristics of the tumor microenvironment and sensitize HCC cells to immunotherapy.

In summary, our study demonstrated that targeting DTX2 attenuates the characteristics of the immunosuppressive microenvironment and sensitizes HCC cells to anti‐PD1 therapy (**Figure** **8**). These findings suggest that DTX2 is a promising immunotherapeutic target in HCC.

## 4 Experimental Section

The detailed methods are described in the supplementary materials.

### Ethics Approval Statement

The study protocol was approved by the Research Ethics Committee of Zhongshan Hospital of Fudan University (Shanghai, China, No.2021BAT4859), and written informed consent was obtained from each patient. All animal experiments were performed with the approval of the Shanghai Medical Experimental Animal Care Committee (No.20220120‐068) and in accordance with the guidelines of the National Academy of Sciences and the National Institutes of Health.

### Patient Consent Statement

All tissues and blood samples were collected after obtaining informed consent in writing from patients or healthy donors.

## Conflict of Interest

The authors declare no conflict of interest.

## Author Contributions

X.W., J.C., Y.C., and S.S. contributed equally to this work. X.‐L. W., J.‐F.C., Y.‐R.C., Y.F., S.‐W. M., Q.‐F. Z., T.‐H. C., F.‐Y. C., Y.‐C. B., and Z.T. performed the experiments; J.G., G.‐Q.Z., S.‐S.S., R.Y., Z.‐Q.G., and W.‐F.Q. analyzed the data; Y.‐R.C., S.‐S.S., and X.‐L.W. wrote the manuscript; J.Z., J.F., W.‐R.L., and Y.‐H.S. supplied the patient samples; J.Z., J.F., W.‐R.L., Y.‐Y.R., and Y.‐H.S. conceived this work. All authors approved the final manuscript.

## Supporting information



Supporting Information

Supporting Information

## Data Availability

All data relevant to the study are available upon reasonable request. Please contact SYH (email: shi.yinghong@zs‐hospital.sh.cn) with any enquiries.
