# Supplementary material for: Targeting Deltex E3 Ubiquitin Ligase 2 Inhibits Tumor‐associated Neutrophils and Sensitizes Hepatocellular Carcinoma Cells to Immunotherapy
Source: Adv Sci (Weinh). 2024 Dec 29;12(7):2408233. doi: 10.1002/advs.202408233 (PMC11831464; doi:10.1002/advs.202408233)
Supplement: Supplementary file 1 — Supporting Information [file ADVS-12-2408233-s002.docx]

**Targeting** **Deltex E3 ubiquitin ligase 2 inhibits** **tumor-associated neutrophils and sensitizes hepatocellular carcinoma cells to immunotherapy**

**Supplementary data**

Xiaoling Wu, Jiafeng Chen, Yiran Chen, Shushu Song, Yuan Fang, Shengwei Mao, Jiafeng Chen, Jun Gao, Guiqi Zhu, Weifeng Qu, Qianfu Zhao, Rui Yang, Zhiqi Guan, Tianhao Chu, Yichao Bu, Yi Wang, Fangyu Chen, Jian Zhou, Jia Fan, Zheng Tang, Weiren Liu, Yuanyuan Ruan, Yinghong Shi

**Methods**

**HCC patients’ samples and clinical information**

The tissue microarray comprises samples from 224 HCC patients who underwent liver resection from 2006 to 2008 in Zhongshan Hospital. Snap-frozen HCC tissues and normal liver tissues were collected from 40 patients on whom the liver resections were performed from October 2021 to January 2022 in Zhongshan Hospital. Human peripheral blood samples were provided by healthy donors. All tissues and blood samples were collected after obtaining informed consent in writing from patients or healthy donors. This study was conducted in accordance with ethical guidelines (Declaration of Helsinki), and approved by the Institutional Review Board.

224 HCC patients who underwent liver resection from 2006 to 2008 in Zhongshan hospital underwent computed tomography or abdominal magnetic resonance imaging scans at an interval of 6 months in the first 2 years after surgery. Serum alpha-fetoprotein (AFP), liver function, and abdominal ultrasound were monitored every 3 months. Patients were followed up until December 2013 and overall survival (OS) was defined as the time between surgery and death or the last follow-up visit. None of these patients received any pre-operative therapy like radiofrequency ablation, anhydrous alcohol injection, TACE or immunotherapy. The histopathological examination of samples was confirmed and diagnosed as HCC after surgery.

**Cell lines and cell culture**

The human hepatoma cell lines (Huh-7, SNU449, SNU387) and mouse hepatoma cell line (Hepa1-6) used in this study were purchased from Shanghai Biological Cell Bank of Chinese Academy of Medical Sciences (Shanghai, China). These cells were cultured in DMEM medium (Gibco, CA, USA) or RPMI 1640 medium (Gibco, CA, USA) containing 10% fetal bovine serum (Tico Europe, Netherlands) and 1% penicillin/streptomycin (Gibco, CA, USA) at 37℃ in 5% CO_2_.

Human-derived primary neutrophils and CD8+ T cells were extracted from human peripheral blood samples. Mice-derived neutrophils and CD8+ T cells were extracted from mouse bone marrow and spleen respectively. All primary immune cells were isolated and used for functional assays immediately. Detailed information will be described in supplementary materials.

For ubiquitination assays, Huh-7 and Hepa1-6 cells were separately treated with 10 μmol/L MG132 (MCE, USA) for 6h. 100nmol/L reparixin was added to culture medium for neutrophil associated experiments *in vitro*. The concentration of selected mDTX2 inhibitor was 5μmol/L for experiments *in vitro*.

For CXCL2 neutralization, 50ng/ml antibody against hCXCL2 or mCXCL2 was added into culture medium for followed experiments. For CXCL6 neutralization, 50ng/ml antibody against hCXCL6 or mCXCL6 (Gcp2) was added into culture medium for followed experiments.

**RNA extraction, reverse transcription, and quantitative real-time PCR (qRT-PCR)**

RNA of tissue samples and cells were extracted by TRIzol Reagent (Invitrogen, USA). RNA concentration and purity were measured by NanoDrop ND2000 (Thermo Scientific, Massachusetts, USA). RNA reverse transcription was performed by HiScript III 1st Strand cDNA Synthesis Kit and Mir-X miRNA First-Strand Synthesis Kit (Takara, USA) according to the instructions. SYBR Green PCR kit (Vazyme, Nanjing, China) was used for qRT-PCR. The CFX96TM Real-time System (Bio-Rad, California, USA) was applied for the reaction. RNA relative expression levels were calculated by 2^-ΔΔCt^ (Ct=threshold cycle). All experiments were performed in triplicate. The primer sequences were listed in Table S1.

**RNA interference and cell transfection**

DTX2 (homo sapiens) and Dtx2 (mus musculus) were interfered by small interfering RNAs (siRNAs). Two siRNAs targeting on DTX2 (homo sapiens) were synthesized by Tsingke Biotech Co (Beijing, China). Three siRNAs targeting on Dtx2(mus musculus) were synthesized by Hanbio Biotechnology Co. (Shanghai, China). The sequences of siRNAs were presented in Table S2. The transfection of siRNAs was accomplished according to the instructions of jetPRIME transfection reagent (Polyplus, France).

**Construction of plasmid, lentivirus package and cell infection**

The DTX2 (homo sapiens) and Dtx2 (mus musculus) gene sequences were synthesized and inserted into pcDNA3.1 plasmid to construct overexpression plasmid (named DTX2 or Dtx2 below) by Hanbio Biotechnology Co. Empty vector was used as the control group (named Vector below). The plasmids of pcDNA-HA-Ub, pcDNA-Flag-DTX2(wild type), pcDNA-Flag-DTX2(Δ1), pcDNA-Flag-DTX2(Δ2), pcDNA-Flag-DTX2(Δ3), pcDNA-Flag-DTX2(Δ4) and pcDNA-Flag-empty vector were synthesized and provided by Hanbio Biotechnology Co. (Shanghai, China). Stably short-hairpin human DTX2 (shDTX2) and stably short-hairpin mouse Dtx2 (shDtx2) were packaged by lentivirus. The viral supernatant was filtered with 0.45 μM syringe filters and further supplemented with 5 µg/ml polybrene to infect target HCC cells. All cell lines underwent selection in the presence of 3 µg/ml puromycin. Cells were harvested 4 days later and followed by qRT-PCR and Western-blot testing the interference efficiency.

**Colony formation assays**

500-1000 cells were cultured evenly in medium on 6-well plates. After 14 days, cells were fixed by 4% paraformaldehyde, followed by 0.1% crystal violet staining (Beyotime Biotechnology, China) for 1h. After washed by PBS for 2 times, the colony spots were counted and imaged.

**Cell viability assays**

Cell Counting Kit (CCK‐8, Yeasen Biotechnology Co., Shanghai, China) was used to measure cell viability capacity. According to the protocol, 100μl culture medium containing 5000 cells was added into each well of the 96-well plate. After 24h, 10μl CCK-8 reagent was added into each well. The 450nm absorbance value was detected with an enzyme labeled analyzer after incubation at 37℃ for 1h.

For IC50 detection of mDTX2 inhibitor, 100μl culture medium containing 1 x 10^4^ Hepa1-6 cells was added into each well of the 96-well plate. On the second day, new 100μl medium was replaced and different concentrations of mDTX2 inhibitors were added. After 24h, 10μl CCK-8 reagent was added into each well. The 450nm absorbance value was detected with microplate reader after incubation at 37℃ for 1h.

**EdU cell proliferation staining**

EdU cell proliferation staining was performed using EdU Cell Proliferation Kit with Alexa Fluor 488 (Beyotime, China). Briefly, 5 × 10^4^ cells were seeded into each well of 6-well plates and cultured for 48h respectively. Subsequently, cells were incubated with 10μm EdU for 2 h, fixed with 4% paraformaldehyde for 15 min, and permeated with 0.3% Triton X-100 for another 15 min. Each well was incubated with 500μl Click Reaction Mixture for 30 min at room temperature in a dark place and then incubated with Hoechst 33342 for 10 min. Finally, cells were observed and imaged by an inverted fluorescence microscope.

**Neutrophil** **Isolation and Culture**

Human neutrophil isolation kit (TBDScience, Tianjin, China) was used to isolate human neutrophils from the peripheral blood of healthy donors. Specifically, human neutrophil isolation solution was slowly added to a 15ml centrifuge tube, followed by the same volume of human peripheral blood. After centrifugation at 600g for 30min, neutrophils were separated into the third layer of the suspension. Then neutrophils were transferred and mixed with 3-5ml of red blood cell lysis buffer (TBDScience, Tianjin, China). After centrifugation at 600g for 10min, cells were cultured in RPMI 1640 medium supplemented with 10% FBS.

Mouse bone marrow neutrophil isolation solution kit (TBDScience, Tianjin, China) was used to isolate mouse neutrophils from bone marrow of C57BL/6 mouse. Briefly, femur and tibia of the euthanized mice were resected with scalpel and washed by 1xPBS for 3 times. Ophthalmic scissors were used to open both ends of the bone. RPMI 1640 medium containing 10% FBS was used to rinse femoral and tibial cavities by 25G needles to obtain bone marrow cell. After centrifuge at 600g for 10min, precipitation of bone marrow cells was obtained. Then neutrophils were isolated from precipitation of bone marrow cells according to the instructions mentioned above.

**Neutrophil Migration Assay**

24-well plates and chambers with 3.0 µm polycarbonate membranes (Corning, USA) were used for neutrophil migration assay. 2×10^5^ neutrophils were resuspended in 100 µl of serum-free RPMI 1640 medium and added to the upper chamber. Supernatants were harvested when HCC cells were cultured for 72h. 600µl RPMI 1640 medium containing 10% FBS alone or with 50% indicated culture supernatants was added to the lower chamber. After incubation in cell incubator for 3 h, cells migrating into the lower chambers were harvested and stained by 0.4% trypan blue (Thermo Fisher, USA). Besides, cells migrating into the lower chambers were harvested and used for RNA extraction and qPCR detection. Countess™ Automatic cell counter (Thermo Fisher, USA) was used for cell counting.

In addition, 2×10^5^ neutrophils were cultured with vehicle medium or indicated medium for 3h. The apoptosis rate of neutrophils was determined using the Annexin V-FITC/PI apoptosis detection kit (Yeasen Biotechnology Co., Shanghai, China). The percentage of Annexin V^+^ PI^-^ and Annexin V^+^ PI^+^ target cells was measured by flow cytometry.

**CD8+ T cell Isolation and Culture**

IPHASE Human CD8+ T Cells Positive Selection Kit (Iphase Biotech, Suzhou, China) was used to extract CD8+ T cells from the peripheral blood of healthy donors. Briefly, human peripheral blood lymphocyte isolation solution (TBDScience, Tianjin, China) was added into a 50ml centrifuge tube. Then equal volume of human peripheral blood was slowly added and centrifuged at 600g for 25min. The second layer of human peripheral blood lymphocytes was transferred to another centrifuge tube. 1× Isolation buffer was added to adjust the cell density to 1×10^8^ cells/ml. IPHASE anti-human CD8 Biotin-Antibody was added to the cell suspension and incubated on ice for 15min. IPHASE CD8+ SA Nanobeads were added and incubated for 15min. Then the centrifuge tube was placed on the magnetic rack to magnetically separate cells for 5min. The isolated CD8+ T cells were cultured in RPMI 1640 medium supplemented with 10% FBS.

Mouse CD8+T cells were isolated from spleen of C57BL/6 mouse. After euthanasia, mice were soaked in 75% alcohol. Sterilized ophthalmic scissors and ophthalmic tweezers were used to dissect spleens and place them in petri dish. The spleen was cut with ophthalmic scissors, and the tissue fragments were repeatedly washed by 1xPBS. Then tissue suspension was filtered twice using a 70um cell strainers and then transferred to a centrifuge tube. The IPHASE Mouse CD8+ T Cells Positive Selection Kit (Iphase Biotech, Suzhou, China) was used to isolate the CD8 T cells according to the instructions mentioned above.

**CD8+ T cell proliferation capacity assay**

The IPHASE Human CD3/CD28 T Cells Activation/Expansion Kit and IPHASE Mouse CD3/CD28 T Cells Activation/Expansion Kit (Iphase Biotech, Suzhou, China) were used to prepare the necessary culture medium for the expansion of CD8+ T cells. The isolated CD8+ T cells were suspended using serum-free RPMI 1640 to a density of 1x10^5^ cells/ml. 1ml cell suspension and 1ml CD8+ T cell expansion medium was added to each well of 6-well plate. Cells were stained by 0.4% trypan blue (Thermo Fisher, USA) and counted by Countess™ Automatic cell counter (Thermo Fisher, USA) every 2-3 days.

CFSE cell proliferation staining was performed using CFDA SE cell Proliferation and Tracer detection kit (Beyotime, China). Briefly, 5×10^6^ CD8+ T cells were mixed with 1ml CFDA SE cell marker solution and 1ml CFDA SE storage solution. Cell suspension was incubated in cell incubator for 10min and then added 10ml RPMI 1640 medium supplemented with 10% FBS. Cell precipitation was collected after centrifuge at room temperature. Cell precipitation was resuspended by CD8+ T cell expansion medium and added to 6-well plates respectively. CFSE staining was detected and recorded using flow cytometry with excitation wavelength of 488nm after 3 days.

**Enzyme-linked immunosorbent assay (ELISA)**

1×10^6^ Huh-7 or Hepa1-6 cells were cultured using DMEM medium with 10% FBS for 1 day. Then cells were replaced with DMEM medium containing 0.5% FBS for 1 day. Culture medium was collected and centrifuged to obtain cell culture supernatant.

Secretion of human CXCL2, CXCL6 and mouse CXCL2, CXCL6 in culture supernatants was examined using Human CXCL2 ELISA Kit (Abcam, USA), Mouse CXCL2 ELISA Kit (Abcam, USA), Human CXCL6 ELISA Kit (Proteintech, China) and Mouse CXCL6 (CXCL5/LIX) ELISA Kit (Proteintech, China) according to manufacturers’ protocol.

**Co-immunoprecipitation (Co-IP) and mass spectrometry (MS)**

Huh-7 was cultured in 10cm diameter culture dishes. Cells transfected with pcDNA-Flag-DTX2, pcDNA-Flag-empty vector or pcDNA-HA-Ub were prepared for co-immunoprecipitation. Cell precipitates were collected and added into 2ml centrifuge tubes. 1ml IP lysis buffer (Beyotime, China) with 1×Protease and Phosphatase Inhibitor Cocktail (MCE, USA) per tube was added to lyse cells for 30min. Pre-washed Protein A/G Magnetic beads (MCE, USA) were added into centrifuge tubes and rotated on disc rotating mixer for 30min subsequently. Then antibodies (anti-Flag, anti-DTX2, anti-H2B) were added and incubated in corresponding tubes for more than 3h at 4℃. Finally, the specific proteins interacting with the target protein were pulled down and adhered to the magnetic beads. These proteins would be separated from the magnetic beads after mixed with 5×SDS-loading buffer and heated up to 96℃ for 10min. Protein sample immunoprecipitated by Flag antibody was sent to Singleronbio (Nanjing, China) for mass spectrometry.

**Western blot**

Tissue samples and cell proteins were extracted by RIPA lysis buffer (Beyotime, Shanghai, China) plus 1×PMSF proteasome inhibitor (Beyotime, Shanghai, China). Protein concentration was measured by BCA kit (Thermo Fisher Scientific, CA, USA). Different proteins were separated in 10%-12.5% SDS-PAGE gel after electrophoresis. Then target proteins were transferred on the polyvinylidene fluoride membranes. After blocked with 5% defatted milk for 1h, the membranes were immersed in primary antibody and incubated overnight at 4℃. Afterwards, membranes were washed and incubated with secondary antibody for 90min at room temperature. ECL Substrate Luminol solution (Bio-Rad, CA, USA) was applied for immunoblot analysis. Detailed information of primary antibody and secondary antibody was presented in Table S3.

**Endogenous and Exogenous** **Ubiquitination Assays**

For endogenous ubiquitination assays, Huh-7 cells were transfected with shDTX2 or shControl packaged by lentivirus and Hepa1-6 were treated with mDTX2 inhibitor (5µM 48h). For exogenous ubiquitination assays, Huh-7 cells transfected with shDTX2 or shControl packaged by lentivirus were transfected with pcDNA-HA-Ub. Cells were collected after transfection for 48h and lysed using IP lysis buffer (Beyotime, Shanghai, China). The subsequent steps were the same as those mentioned-above for Co-IP.

**Chromatin immunoprecipitation quantitative PCR assays (ChIP-qPCR)**

ChIP-qPCR was performed using ChIP kit (abcam) according to its instruction. Briefly, Chromatin from 3×10^6^ cells was used for each ChIP experiment. H2BK120ub1, H3K4me3 antibodies and IgG isotype were used. The final DNA extracts were amplified with qPCR. The values from the immunoprecipitated samples were normalized to those from the input DNA. The primer sequences were listed in Table S3.

**Screening of mouse DTX2 inhibitors (mDTX2i)**

Mouse DTX2 structure was searched and downloaded from Alphafold website (https://alphafold.ebi.ac.uk/entry/Q8R3P2). Protein structure was optimized with the Protein Preparation Wizard panel (Schrödinger 2021) by correcting the bond order, adding hydrogen atoms, distributing charges, and predicting the protonation states (pH 7.0). The OPLS4 force field was used for constrained energy optimization to eliminate the atomic conflict on the structure with RMSD of heavy atoms converges to 0.3 Å, and the side chain position was optimized to obtain a reasonable side chain structure.

The compounds from ChemDiv library were processed with the LigPrep panel (Schrödinger 2021). Firstly, compounds were protonated and desalted with Epik program at pH7.0 ± 2.0 to generate tautomers and maintain the original atomic chirality. In order to ensure the conformational diversity of small molecules in the virtual screening process, each small molecule was generated up to 32 conformations.

Glide HTVS [high throughput virtual screening mode] → Glide SP [standard precision mode] was used for screening. The protein structure was set as rigid, small molecules as flexible, and other parameters as default values. Docking results were carried out energy optimization, and top 10% with docking scoring shall be reserved for the next screening.

Protein ligand interface fingerprint (PLIF) was used to calculate and analyze the binding sites and interaction types of compounds and mDTX2 protein structure. The hydrogen bond, ionic bond, surface contact energy and other interactions were described and used to exclude the docking poses with low concurrency.

The drug properties such as water solubility (logS), lipid water distribution coefficient (logP), molecular weight, molecular flexibility, hydrogen bonding properties, surface accessibility area (TPSA) and other indicators of the compounds were calculated and analyzed by Stardrop software (Version 6.5.0)) (https://www.optibrium.com/stardrop/).

Surface-plasmon resonance experiments were performed at 25 °C on a BIAcore T200 using CM5 sensor chips, and data were analysed using BIAcore T200 Evaluation software (GE Healthcare) following the manufacturer’s instruction. In brief, a cell on the CM5 sensor chip was activated with a mixture of 200μM 1-ethyl-3-(3-dimethylaminopropyl) carbodiimide (EDC) and 50μM N-hydroxysuccinimide (NHS) at 10μl/min for 420s. A total of 50μl of protein by mixing with 180 μl of 10 mM sodium acetate solution, pH 5.0, was then immobilized on the surface of the cell at 10 μl/min for 420s for two repetitive runs. The cell was then blocked with 1 M ethanolamine (10μl/min for 420s). A neighbouring aisle that served as a reference was similarly activated and blocked, except that PBS adjusted to pH 5.0 was used for immobilization. Both of the aisles were then equilibrated with PBS. Molecule stock solution was diluted to a series of concentrations in PBS, and was flowed at 10μl/min for 150s in each run. At the end of each flow, cells were regenerated for 5min with 10mM glycine-HCl (pH 2.0) solution at 10μl/min. Data from the sample cell were collected using BIAcore T200 Control software (v. 2.0, GE Healthcare), and were subtracted by those from the reference cell. Association and dissociation constants were obtained by global fitting of the data to a 1:1 Langmuir binding model using BIAcore T200 Evaluation software (v.2.0, GE Healthcare). Data were exported to Origin 7 software (v.7.0552, OriginLab) for generating the final figures.

**Immunohistochemical staining (IHC) and multiplex immunofluorescence**

All samples and tissue microarray needed for IHC or multiplex immunofluorescence staining were stored in 4% paraformaldehyde. Tissue slices were obtained by dehydration, paraffin embedding and sectioning. Subsequently, the slices were stained with corresponding antibody. The images were generated by automatic digital scanning and analysis system (Aperio VERSA 8, Germany) and quantified by Aperio ImageScope software (Leica Biosystems).

For multiplex immunofluorescence, the paraffin sections of tissue slices were removed and placed in a repair box with PH6.0 citric acid repair buffer at 95°C for 20min. After natural cooling, the slices were washed in PBS (PH7.4) and then placed in 3% hydrogen peroxide solution for 15min incubation at room temperature away from light. The tissue was uniformly covered with 3%BSA at room temperature for 60min. After that, primary antibody diluted with antibody diluent was added to the slices in a wet box sheltered away from light at 4°C and incubated overnight. Then tissue slices were covered with second antibody and incubated at room temperature for 60min. Multicolor immunofluorescence kit (RecordBio, Shanghai, China) was used to add fluorescent dye for 10-15min reaction. Then repeat the above operation for different markers needed in the experiment. Finally, DAPI dye solution was added to the slices and incubated at room temperature away from light for 10min. The slices were slightly dried and sealed with anti-fluorescence quenching buffer.

**Mass cytometry**

Mouse subcutaneous tumors were removed from euthanized mice and put into tissue storage solution. Tumor tissues were washed twice using cell culture medium and then were cut into 1mm^3^ pieces. Collected these pieces into indicated tube and added digestive enzyme mix according to the instructions (Miltenyi Biotec-mouse Tumor Dissociation Kit). Then tissue suspension was filled with cell culture medium up to 5 mL and incubated in a shaking incubator at 37℃ for 1 hour. Single cell suspension was filtrated through the 70μm cell strainer. Cells were collected after centrifugation at 300g for 5 minutes at 2-8°C and resuspended for further staining. For mass cytometry analysis, purified antibodies (CD45, CD3e, CD8a, CD4, TCRb, TCRgd, CD25, FOXP3, CD19, B220, NK1.1, CD49b, CD11b, Ly6G, Gr1, CD64, Ly6C, F4/80, CD11c, MHCII, CD44, CD62L, CD127, T-bet, CD206, iNOS, CD103, CD69, CD27, PD1, MERTK, CD172a, ICOS, Tim3, Granzyme B, Ki67, CXCR3, CCR4, CCR2, CCR6) were purchased from BioLegend, eBioscience, BioXcell, R&D systems and BD Biosciences. Antibody labeling with the indicated metal tag was performed using the MaxPAR antibody Labelling kit (Fluidigm). All detailed information were presented in Table S6. Conjugated antibodies were titrated for optimal concentration before use.

Cells were washed once with 1×PBS and then stained with 100μl of 250nM cisplatin (Fluidigm) for 5min on ice to exclude dead cells, and then incubated in Fc receptor blocking solution before stained with surface antibodies cocktail for 30min on ice. Cells were washed twice with FACS buffer (1×PBS+0.5%BSA) and fixed in 200μl of intercalation solution (Maxpar Fix and Perm Buffer containing 250nM 191/193Ir, Fluidigm) overnight. After fixation, cells were washed once with FACS buffer and then perm buffer (eBioscience), stained with intracellular antibodies cocktail for 30 min on ice. Cells were washed and resuspend with deionized water, adding into 20% EQ beads (Fluidigm), acquired on a mass cytometer (Helios, Fluidigm).

Data of each sample were debarcoded from raw data using a doublet-filtering scheme [1] with unique mass-tagged barcodes. Each .fcs file generated from different batches was normalized through bead normalization method[2]. Manually gate data was achieved by FlowJo software to exclude to debris, dead cells and doublets, leaving live, single immune cells. Apply the X-shift clustering algorithm to all cells to partition the cells into distinct phenotypes based on marker expression levels[3]. Annotate cell type of each cluster according to its marker expression pattern on a heatmap of cluster vs marker. Use the dimensionality reduction algorithm t-SNE to visualize the high-dimensional data in two dimensions and show distribution of each cluster and marker expression and difference among each group or different sample type[4]. Perform T-test statistical analysis on the frequency of annotated cell population.

**Flow cytometry**

Cells from subcutaneous tumor were prepared by the procedure mentioned above, and then blocked with Fc block (anti-mouse CD16/32, BioLegend) on ice for 30min. Following antibodies were used for lymphoid and myeloid immune cells staining: anti-mouse CD45, anti-mouse CD3, anti-mouse CD4, anti-mouse CD8, anti-mouse NK-1.1, anti-mouse F4/80, anti-mouse CD11b, anti-mouse CD11c, anti-mouse Ly6G, anti-mouse MHC-II, anti-mouse CD25 and anti- mouse PD1. Detailed information on antibodies were presented in Table S5. Cells were fixed, permeabilized, and stained with anti-mouse Foxp3, anti-mouse Granzyme B, anti-mouse CD206 and anti-mouse Arg1. For staining of TNFα and IFNγ, cells were incubated with a Cell Stimulation Cocktail (Invitrogen) and stained with anti-mouse IFNγ or anti-mouse TNFα after fixation/permeabilization. CD45+ CD11b+ Ly6G+ cells were identified as neutrophils for qPCR.

For the functional assessment of human or mouse neutrophils, cells were stained with anti-ARG1. Tumor-associated neutrophils infiltrated in orthotopic tumors were obtained by flow cytometry sorting of CD45+ CD11b+ Ly6G+ cells in tumor tissues. For human CD8+ T cells, cells were incubated with a Cell Stimulation Cocktail (Invitrogen) and stained with anti-IFNγ after fixation/permeabilization.

**RNA-sequencing**

Cells were harvested and total RNA was extracted by using RNAmini kit (Qiagen, Germany). Enrichment of mRNA, fragmentation, reverse transcription, library construction, Illumina Novaseq 6000 and data analysis were performed by Genergy Biotechnology Co. Ltd. (Shanghai, China).

**Cleavage Under Targets and Tagmentation (CUT&Tag)**

CUT&Tag assay was performed as described previously with modifications [5]. Briefly, 1×10^6^ cells were washed twice gently with wash buffer (20mM HEPES pH 7.5; 150mM NaCl; 0.5mM Spermidine; 1×Protease inhibitor cocktail). 10μl Concanavalin A coated magnetic beads (Bangs Laboratories) were added per sample and incubated at room temperature for 10min. Unbound supernatant and resuspended bead-bound cells were removed with dig wash buffer (20mM HEPES pH 7.5; 150mM NaCl; 0.5mM Spermidine; 1×Protease inhibitor cocktail; 0.05% Digitonin; 2mM EDTA). A 1:50 dilution of H2K120ub1 or H3K4me3 or H3K79me3 antibody was added and incubated on a roating platform overnight at 4°C. Then primary antibody was removed using magnet stand. Sencondary antibody (Anti-Rabbit IgG antibody, Goat monoclonal, Millipore AP132) was diluted 1:100 in dig wash buffer and cells were incubated at RT for 60min. Cells were washed using the magnet stand 2-3 times in dig wash buffer. A 1:100 dilution of pA-Tn5 adapter complex was prepared in dig-med buffer (0.01% Digitonin; 20mM HEPES pH 7.5; 300mM NaCl; 0.5mM Spermidine; 1× Protease inhibitor cocktail) and incubated with cells at RT for 1h. Cells were washed for 5min in 1 ml dig-med buffer. Then cells were resuspended in tagmentation buffer (10mM MgCl_2_ in dig-med Buffer) and incubated at 37°C for 1h. DNA was purified using phenol-chloroform-isoamyl alcohol extraction and ethanol precipitation.

To amplify libraries, 21μl DNA was mixed with 2μl of a universal i5 and a uniquely barcoded i7 primer. A volume of 25μl NEBNext HiFi 2×PCR Master mix was added and mixed. The sample was placed in a Thermocycler with a heated lid using the following cycling conditions: 72°C for 5mins (gap filling); 98°C for 30s; 14 cycles of 98°C for 10s and 63°C for 30s; final extension at 72°C for 1 min and hold at 8°C. Libraried clean-up was performed XP beads (Beckman Counter).

Raw data (raw reads) of fastq format were firstly processed through in-house perl scripts. In this step, clean data (clean reads) were obtained by removing reads containing adapter, reads containing ploy-N and low-quality reads from raw data. At the same time, Q20, Q30 and GC content the clean data were calculated. All the downstream analyses were based on the clean data with high quality. Before read mapping, clean reads were obtained from the raw reads by removing the adaptor sequences. The clean reads were then aligned to reference genome sequences using the bwa program. The bam file generated by the unique mapped reads as an input file, using macs2 software for callpeak with cutoff q-value < 0.05. The HOMER's findMotifsGenome.pl tool was used for Motif analysis. The input file is the peak file and the genome fasta file. The DNA sequence is extracted according to the peak file, and the sequence is compared with the Motif database to obtain the Motif. Peaks were annotated by using homer's annotatePeaks.pl. Count the results of the annotations and plot the distribution results using R.

**Assay for Transposase Accessible Chromatin with high-throughput sequencing (ATAC-seq)**

ATAC-seq was performed according to ATAC-seq protocol by Shanghai Jiayin Biotechnology Ltd. In brief, cells were harvested from cell culture and lysed in lysis buffer. The Nextera DNA Library Preparation Kit (Illumina) was used to perform the transposition according to the manufacturer’s manual. 50000 nuclei were pelleted and resuspended with transposase, for 30 minutes at 37°C. The transposed DNA fragments were purified immediately after with a MinElute PCR Purification Kit (Qiagen). After samples were PCR-amplified using 1×NEBNext High-Fidelity PCR Master Mix (New England Biolabs, MA). Subsequent libraries were purified with the MinElute PCR Purification Kit (Qiagen) and subjected to sequencing on Illumina Novaseq 6000 using PE150.

Raw data (raw reads) of fastq format were firstly processed through in-house perl scripts. In this step, clean data (clean reads) were obtained by removing reads containing adapter, reads containing ploy-N and low-quality reads from raw data. At the same time, Q20, Q30 and GC content the clean data were calculated. All the downstream analyses were based on the clean data with high quality. Before read mapping, clean reads were obtained from the raw reads by removing the adaptor sequences. The clean reads were then aligned to reference genome sequences using the bwa program. The bam file generated by the unique mapped reads as an input file, using macs2 software for callpeak with cutoff q-value<0.05. The HOMER's findMotifsGenome.pl tool was used for Motif analysis. The input file is the peak file and the genome fasta file. The DNA sequence is extracted according to the peak file, and the sequence is compared with the Motif database to obtain the Motif. Peaks were annotated by using homer's annotatePeaks.pl. Count the results of the annotations and plot the distribution results using R.

**Animal models construction**

6-week-old male C57BL/6 and 6-week-old male non-obese diabetic/severe combined immune deficiency (NOD/SCID) mice were purchased from the Shanghai Laboratory Animal Center of the Chinese Academy of Sciences. Experimental operations and raising of mice were completed in the department of laboratory animal science of Fudan University Fenglin Campus. All experiments were performed with the approval of the Shanghai Medical Experimental Animal Care Committee and in accordance with the guidelines of the National Academy of Sciences and the National Institutes of Health.

6-week-old male C57BL/6 and non-obese diabetic/severe combined immune deficiency (NOD/SCID) mice were purchased from the Shanghai Laboratory Animal Center of the Chinese Academy of Sciences. Experimental operations and raising of mice were completed in the department of laboratory animal science of Fudan University Fenglin Campus. All experiments were performed with the approval of the Shanghai Medical Experimental Animal Care Committee and in accordance with the guidelines of the National Academy of Sciences and the National Institutes of Health.

For subcutaneous tumor model, 6-week-old male C57BL6 or NOD/SCID mice were randomly caged for raising. Each group contains 6 mice. 2×10^6^ Hepa1-6 shControl, shDtx2, Hepa1-6 Dtx2 or Hepa1-6 Vector cells were injected into the right posterior flanks of mice. Mice were euthanized after 28 days of tumor growth. Subcutaneous tumors were measured using calipers and weighted. Tumor volumes were estimated by another researcher from two perpendicular measurements using the formula V =0.5 × L × W^2^.

For the exhaustion models of neutrophils, CD8+ T cells and CD4+ T cells, anti-mouse Ly6G (Bioxcell), anti-mouse CD8a (Bioxcell), anti-mouse CD4 (Bioxcell) and IgG2a (Bioxcell) were used. 6-week-old male C57BL6 mice were randomly caged for raising. Each group contains 5 mice. Two days before tumor implantation using Hepa1-6 cells, mice were treated with intraperitoneal injection of an initial dose of 200μg/mouse of antibodies in PBS, followed by similar dosing with 100μg/mouse every 3 days throughout the course of tumor growth. Mice were euthanized after 28 days of tumor growth. Subcutaneous tumors were measured using calipers and weighted. Tumor volumes were estimated by another researcher from two perpendicular measurements using the formula V =0.5 × L × W^2^.

For treatment model, 6-week-old male C57BL6 mice were randomly caged for raising. Each group contains 5 mice. Mice were injected with Hepa1-6 cells and waited 1 week for tumor growth. After one week, the indicated drug (reparixin or PD1 mAb) was injected intraperitoneally every 3 days. The indicated drug (Chemdiv Num: C548-0038, Y043-4427 or Y501-5662) was injected subcutaneously every 3 days. The doses of different drugs were 50μg for reparixin (MCE, USA), 200μg for PD1 mAb (Bioxcell), 200μg for small molecular compounds. Mice were euthanized after 28 days of tumor growth. Subcutaneous tumors were measured using calipers and weighted. Tumor volumes were estimated by another researcher from two perpendicular measurements using the formula V =0.5 × L × W^2^. In order to evaluate the survival time of mice bearing tumor, mice were euthanized and the overall survival time was recorded when the length of subcutaneous tumor exceeded 2cm.

For spontaneous HCC model, the pT3-β-catenin-N90-luc was constructed and amplified by Hanbio Biotechnology Co. 10 μg of pX330-p53 (Addgene 59910), 10 μg pT3-β-catenin-N90-luc and 10 μg of CMV-SB13 Transposase were resuspended in sterile normal saline solution. 2 mL of this solution were injected into the lateral tail vein of 6-week-old C57BL/6 J mice in 6-8 s. After 1 week, mice were injected intraperitoneally with PD1 mAb (200μg) or mDTX2i (200μg) every 3 days. The bioluminescence imaging was performed 3 weeks after HDTVi. The survival time was recorded until the mice died.

For orthotopic tumor model, 6-week-old male C57BL6 mice were randomly caged for raising. Each group contains 3 mice. Mice were fixed on a sterile operating table after intravenous anesthesia. Sterile ophthalmic scissors were used to cut 1cm incision at the median abdominal line to expose the abdominal cavity. Hepa1-6 cells were mixed in FBS-free DMEM medium and Matrigel (Yeasen Biotechnology Co., Shanghai, China) at 2:1 volume. 1×10^6^ cells were extracted and injected into the left lobe of the mouse liver with a 30G needle. After hemostasis, the abdomen was closed using medical absorbable suture. After 3 days, mice were injected intraperitoneally with PD1 mAb (200μg) or mDTX2i (200μg) every 3 days. Mice were euthanized 14 days later. To evaluate the side effects of mDTX2i and PD1 mAb, retro-orbital blood collection in mice was used. Orthotopic transplantation tumor was isolated from the left lobe of liver and weighted by another researcher.

**Data resource and analysis**

The Cancer Genome Atlas (TCGA) liver hepatocellular carcinoma (LIHC) database was used for the evaluation on different gene expression and comparison of overall survival and recurrence-free survival, which could be found in GEPIA website (http://gepia.cancer-pku.cn/index.html) and TIMER website (https://cistrome.shinyapps.io/timer/). Besides, TIMER website was used to predicate the correlation between different genes and immune infiltration level. Estimation of stromal and immune cells (ESTIMATE score) in HCC tissues from TCGA database were downloaded from https://bioinformatics.mdanderson.org/estimate/index.html. CIBERSORT immune infiltration prediction was accomplished using TCGA LIHC database. Pearson’s correlation analysis was performed by R to determine the correlation between two variables.

**Statistical analysis**

Experimental results were shown as mean ± SDs. According to whether the data conforms to the normal distribution, difference between 2 groups were compared using Student’s t test or non-parametric tests. Differences between multiple groups were compared using two-way analysis of variance. The Kaplan-Meier method and the long-rank test was applied for survival curve comparison. All functional experiments *in vitro* were performed triplicates. Significant differences in statistics are denoted with *(p < 0.05), **(p < 0.01), ***(p < 0.001) and **** (p < 0.0001) in figures. All statistical analyses were performed using SPSS 22.0 and GraphPad Prism 8.0 software.

**Supplementary Figures and Legends**


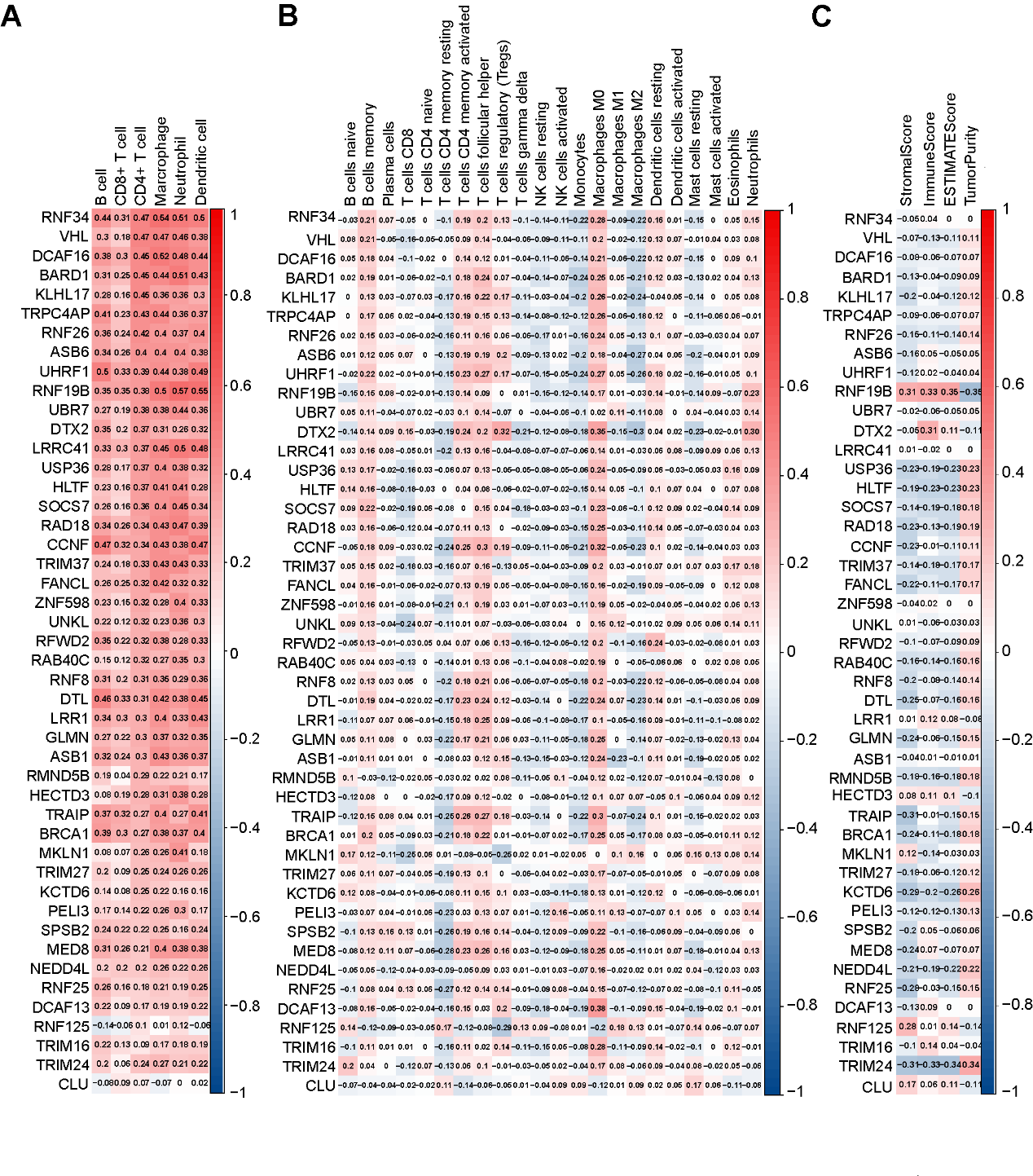


**Supplementary Figure S1. E3 ligases screened by TIMER, CIBERSORT and ESTIMATE tools.**

(A) The correlation between immune cell infiltration in HCC and expression of E3 ligases. Data analyzed from the TCGA LIHC dataset by TIMER web server. (B) The correlation between immune cell infiltration in HCC and expression of E3 ligases. Data analyzed from the TCGA LIHC dataset by CIBERSORT algorithm. (C) The correlation between stromal score or immune score and expression of E3 ligases. Data analyzed from the TCGA LIHC dataset by ESTIMATE tool. TIMER, Tumor IMmune Estimation Resource; ESTIMATE, Estimation of STromal and Immune cells in MAlignant Tumor tissues using Expression data.


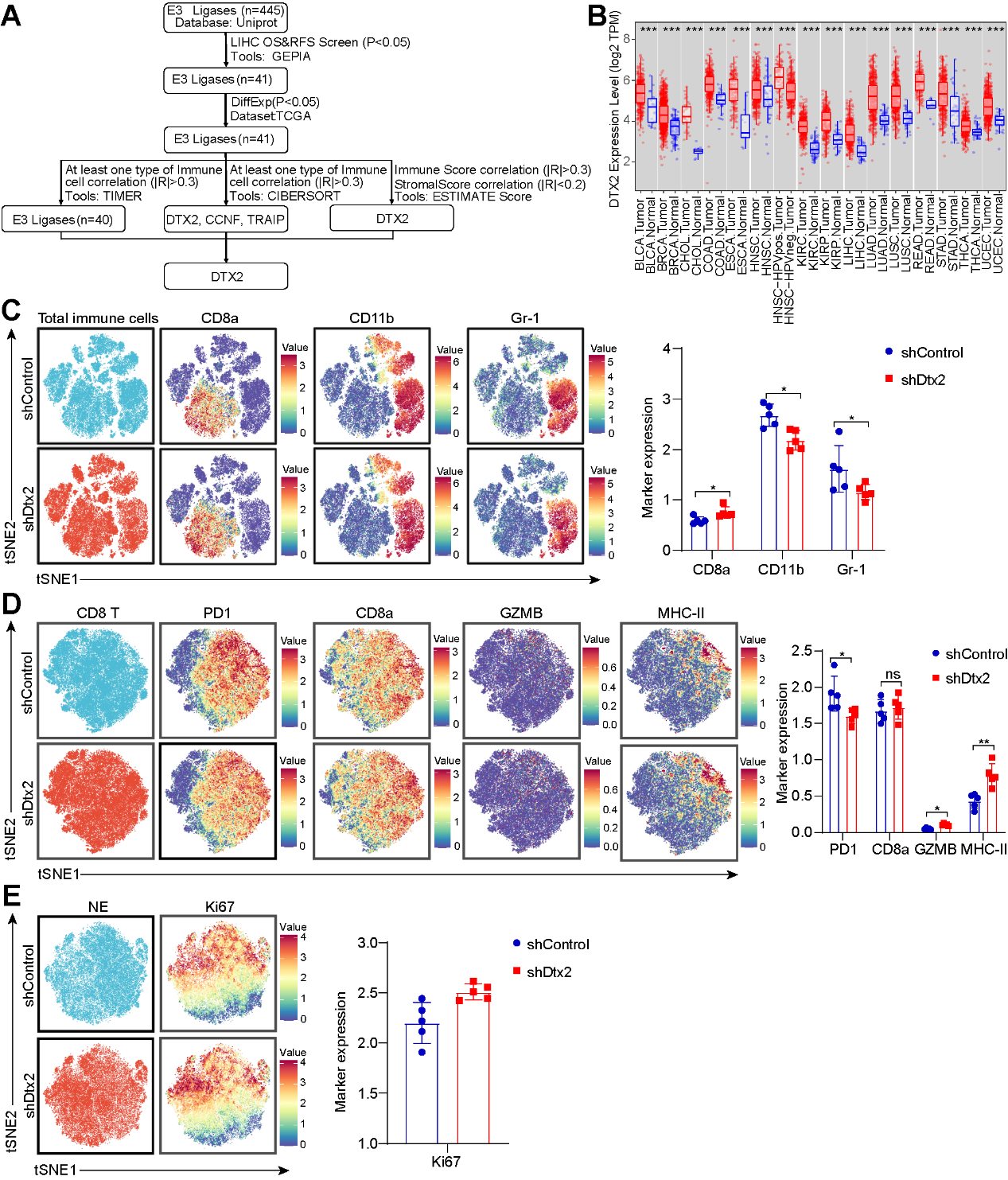


**Supplementary Figure S2. DTX2 in HCC promotes the infiltration of TANs and inhibits the infiltration of CD8+ T cell.**

(A) Flow chart of E3 ligases screening. (B) The expression difference of DTX2 in various tumor tissues and normal tissues obtained from TIMER website. (C) tSNE dimensionality reduction clustering diagram of CD8a, CD11b, and Gr-1 expressions of infiltrating immune cells (n=5 per group). (D) tSNE dimensionality reduction clustering diagram of PD1, CD8a, GZMB and MHC-II expressions of infiltrating CD8+ T cells (n=5 per group). (E) tSNE dimensionality reduction clustering diagram of Ki67 expression of infiltrating neutrophils (n=5 per group). Data are presented as the mean ± SDs. *p < 0.05.


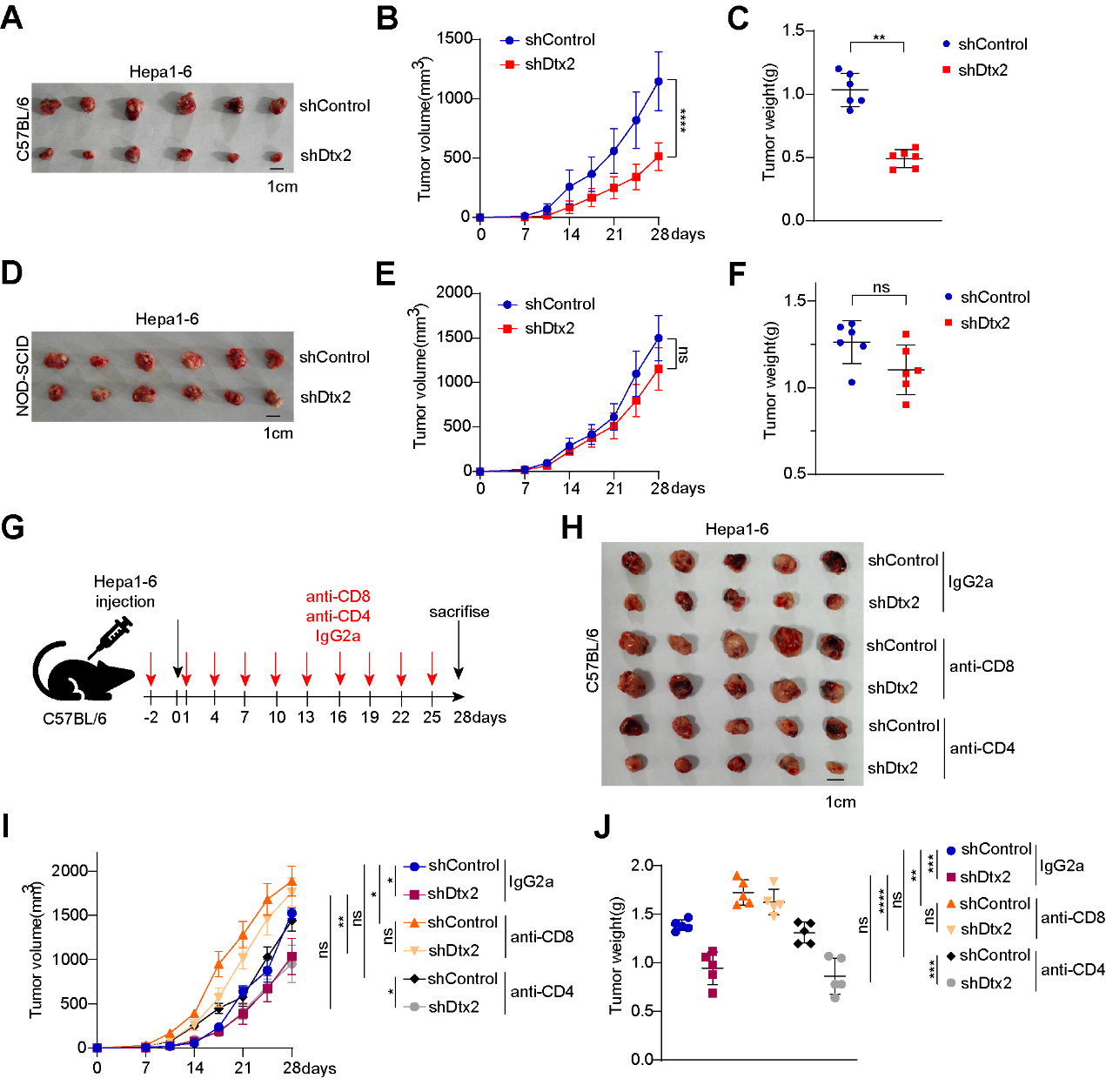


**Supplementary Figure S3. DTX2 promotes tumor growth by affecting the immune microenvironment.**

(A-C) Tumor image (A), tumor growth curve (B) and tumor burden (C) of subcutaneous tumors constructed in C57BL/6 mice (n=6 per group). (D-F) Tumor image (D), tumor growth curve (E), and tumor burden (F) of subcutaneous tumors constructed in NOD/SCID mice (n=6 per group). (G) Schematic diagram of subcutaneous tumor construction with the depletion of CD4+ T cells or CD8+ T cells. (H-J) Tumor image (H), tumor growth curve (I) and tumor burden (J) of subcutaneous tumors constructed by Hepa1-6 shControl or Hepa1-6 shDtx2 treated with IgG2a or anti-CD8 or anti-CD4 respectively (n=5 per group). Data are presented as the mean ± SDs. *p < 0.05, **p < 0.01, ***p < 0.001，****p < 0.0001. NOD-SCID, non-obese diabetic-severe combined immune deficiency; ns, nonsignificant difference.


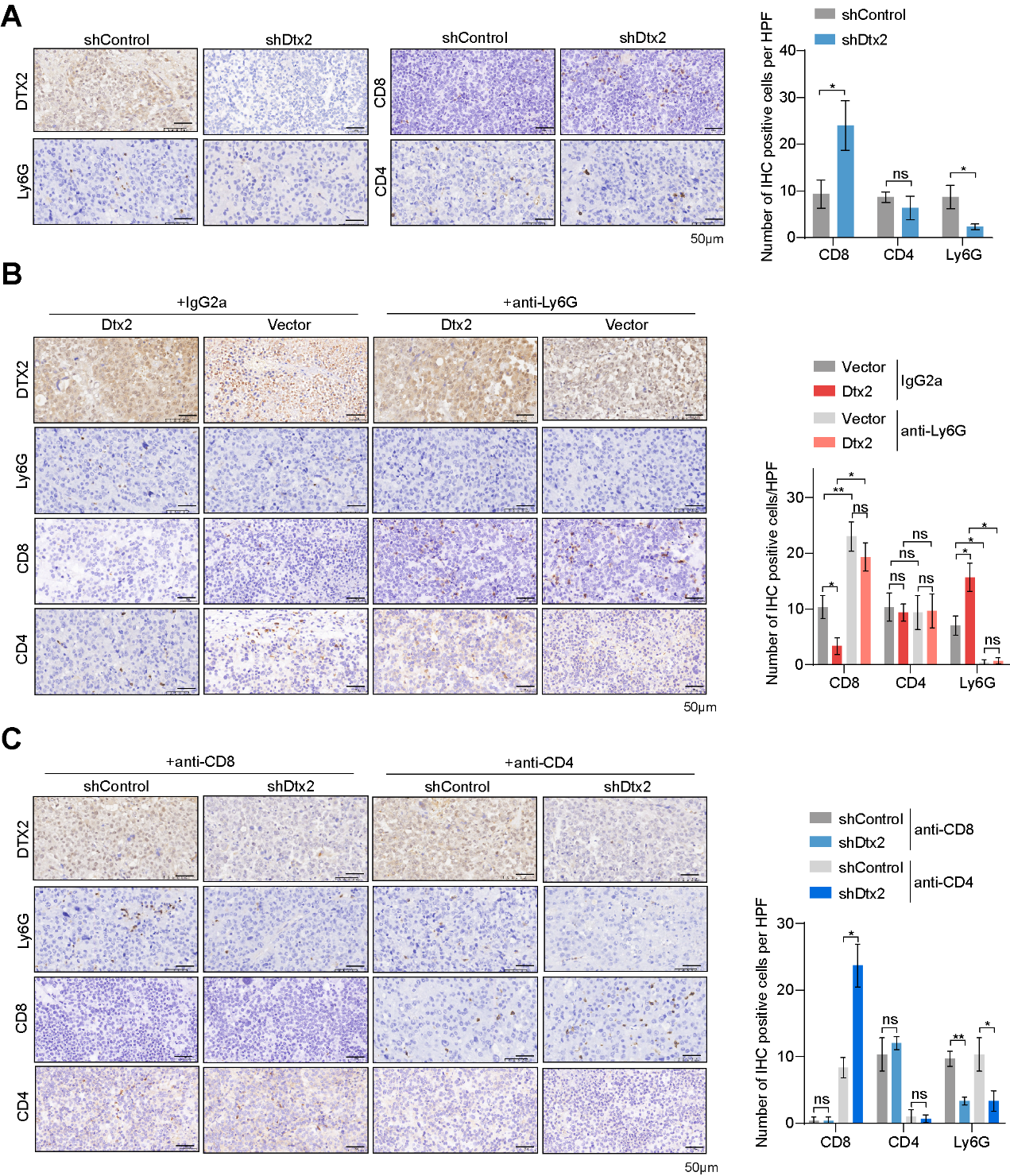


**Supplementary Figure S4. IHC staining of subcutaneous tumors from C57BL/6 mice**

(A) The IHC staining image and statistical chart of DTX2, Ly6G, CD8, and CD4 in subcutaneous tumors constructed with Hepa1-6 shControl cells or Hepa1-6 shDtx2 cells. (B) The IHC staining image and statistical chart of DTX2, Ly6G, CD8, and CD4 in subcutaneous tumors constructed with Hepa1-6 Vector cells and Hepa1-6 DTX2 cells treated by IgG2a or anti-Ly6G. (C) The IHC staining image and statistical chart of DTX2, Ly6G, CD8, and CD4 in subcutaneous tumors constructed with Hepa1-6 shControl cells or Hepa1-6 shDtx2 cells treated by anti-CD8 or anti-CD4. Data are presented as the mean ± SDs. *p < 0.05, **p < 0.01. HPF, HighPowerField; ns, nonsignificant difference.


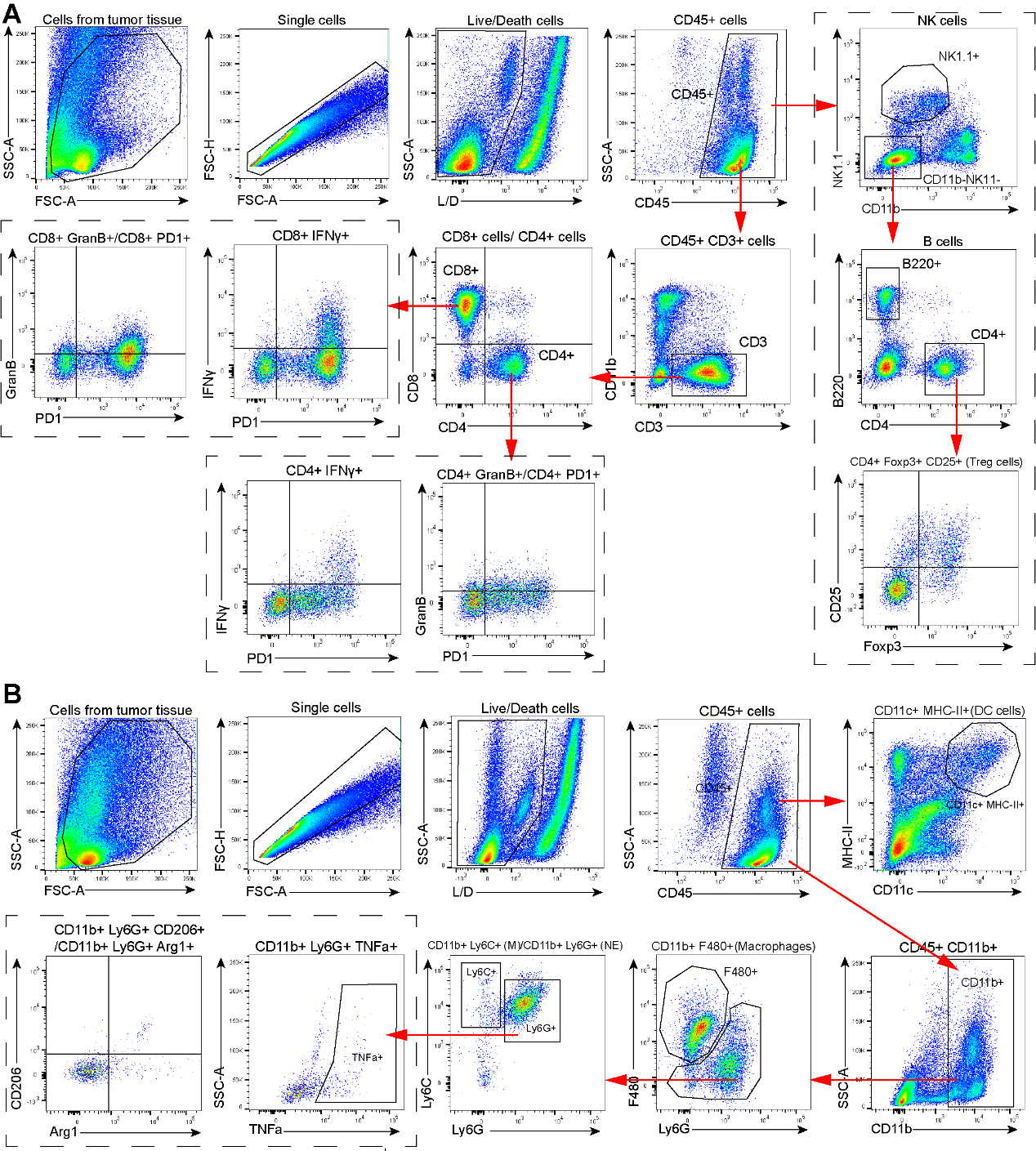


**Supplementary Figure S5. Gating strategy for flow cytometry analysis of infiltrating immune cells in subcutaneous tumors.**

(A) Flow cytometry representative panel of lymphoid cells: NK cells (CD45+ NK1.1+), B cells (CD45+ B220+), CD8+ T cells (CD45+ CD3+ CD8+), CD4+ T cells (CD45+ CD3+ CD4+), Treg cells (CD45+ CD3+ CD4+ CD25+ Foxp3+), GZMB+ CD8+ T cells (CD45+ CD3+ CD8+ GranB+), IFNγ + CD8+ T cells (CD45+ CD3+ CD8+ IFNγ +), PD1+ CD8+ T cells (CD45+ CD3+ CD8+ PD1+). (B) Flow cytometry representative panel of myeloid cells: dendritic cells (CD45+ CD11c+ MHC-II+), macrophages (CD45+ CD11b+ F480+), neutrophils (CD45+ CD11b+ Ly6G+), monocytes (CD45+ CD11b+ Ly6C+), TNFα + neutrophils (CD45+ CD11b+ Ly6G+ TNFα +), ARG1+ neutrophils (CD45+ CD11b+ Ly6G+ ARG1+). NK, nature killer; DC, dendritic cell; GranB, granzyme B; IFNγ, interferon-γ; ARG1, arginase1; TNFα, tumor necrosis factor-α.


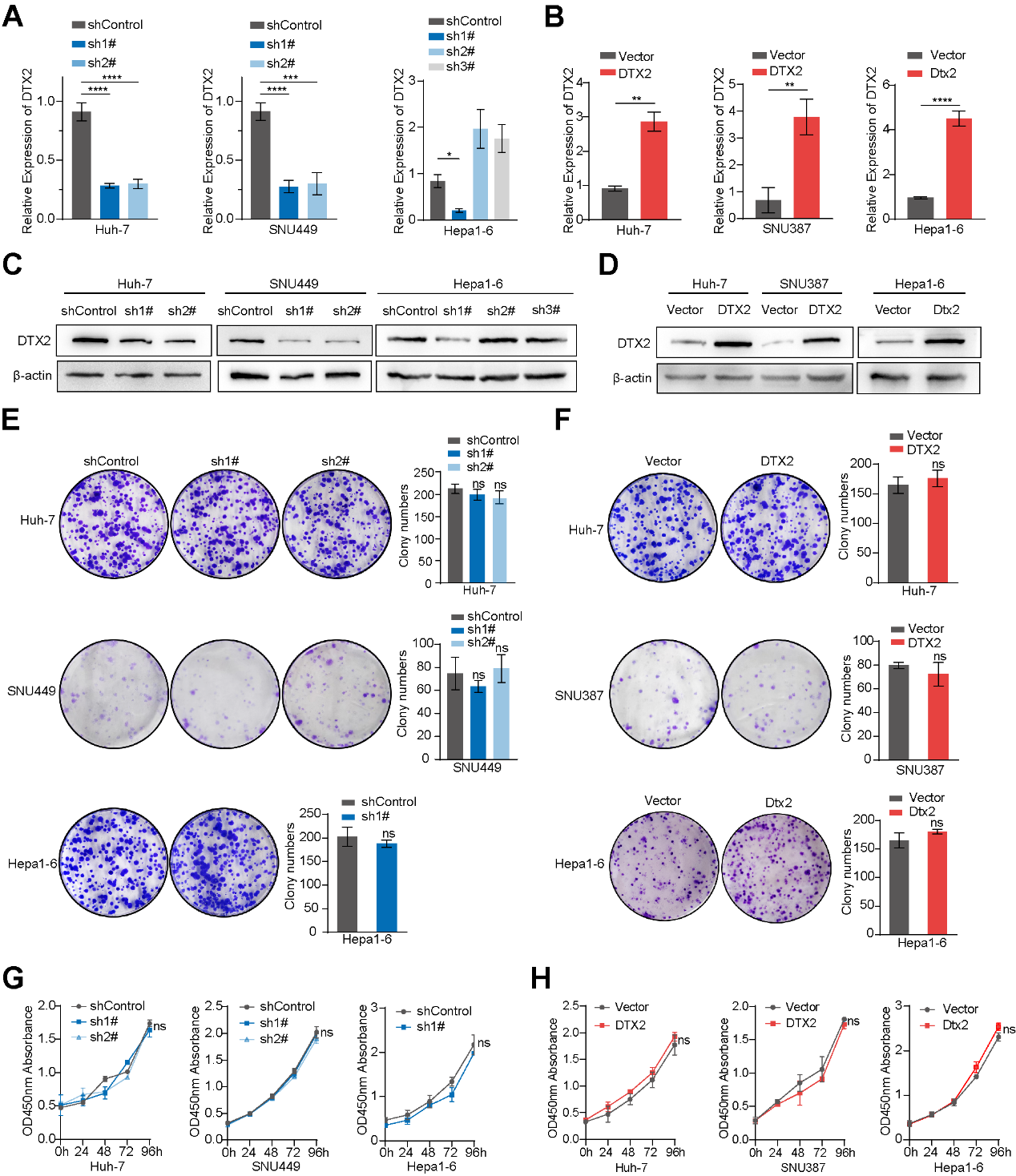


**Supplementary Figure S6. Knockdown or overexpression of DTX2 in HCC did not affect tumor growth ability *in vitro.***

(A) The knockdown efficiency of shDTX2 verified by qPCR. (B) The overexpression efficiency of DTX2 verified by qPCR. (C) The knockdown efficiency of shDTX2 verified by Western blot. (D) The overexpression efficiency of DTX2 verified by Western blot. (E) Colony formation diagram of shControl group and shDTX2 group. (F) Colony formation diagram of Vector group and DTX2 overexpression group. (G) CCK-8 assay of shControl group and shDTX2 group. (H) CCK-8 assay of Vector group and DTX2 overexpression group. Data are presented as the mean ± SDs. *p < 0.05, **p < 0.01, ****p < 0.0001. ns, nonsignificant difference.


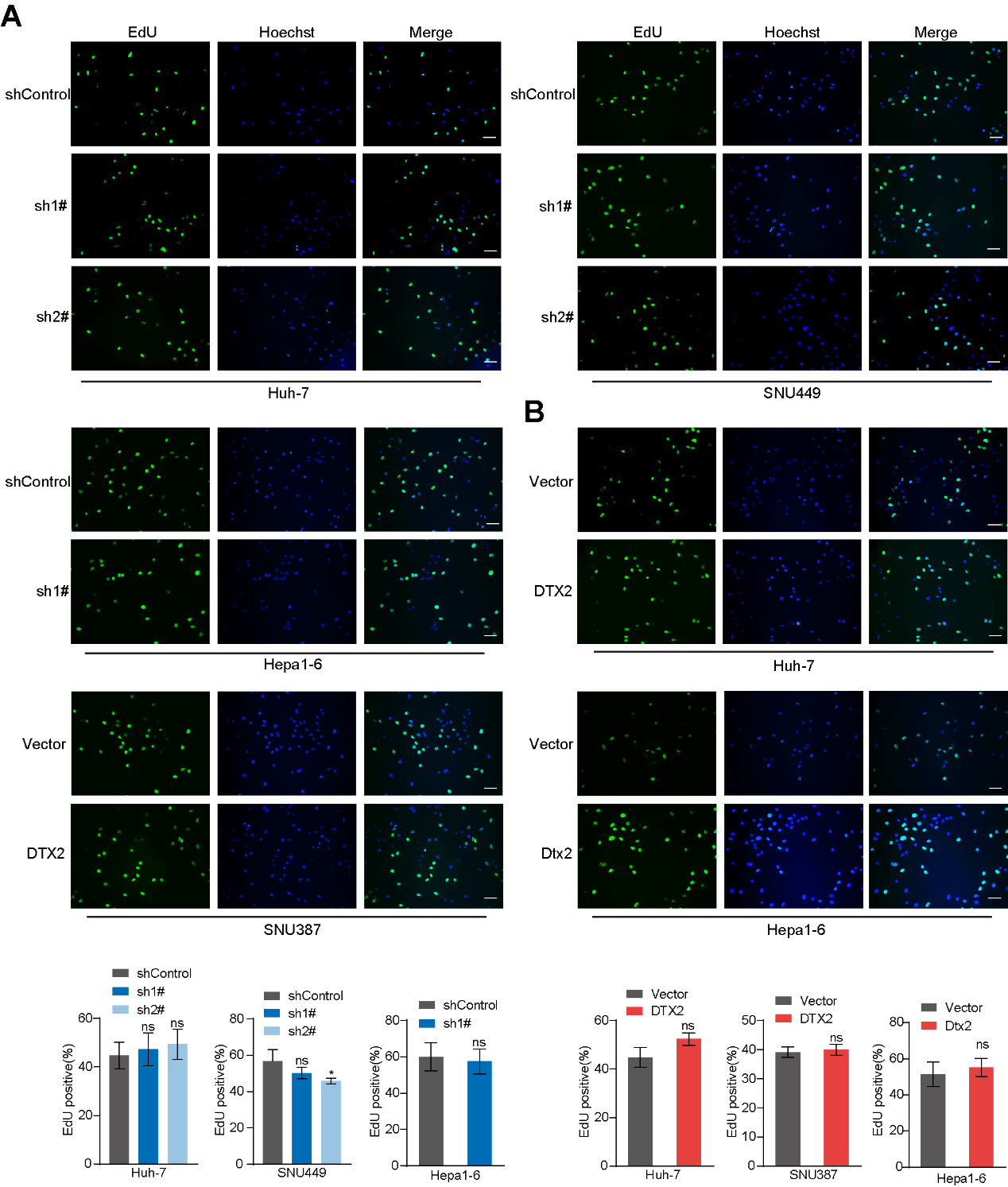


**Supplementary Figure S7. EdU staining of HCC cells with knockdown or overexpression of DTX2.**

(A) EdU staining image and statistical charts of Huh-7, SNU449 and Hepa1-6 cells with shControl group and shDTX2 group. (B) EdU staining image and statistical charts in Huh-7, SNU387 and Hepa1-6 cells with Vector group and DTX2 overexpression group. Data are presented as the mean ± SDs. ns, nonsignificant difference.


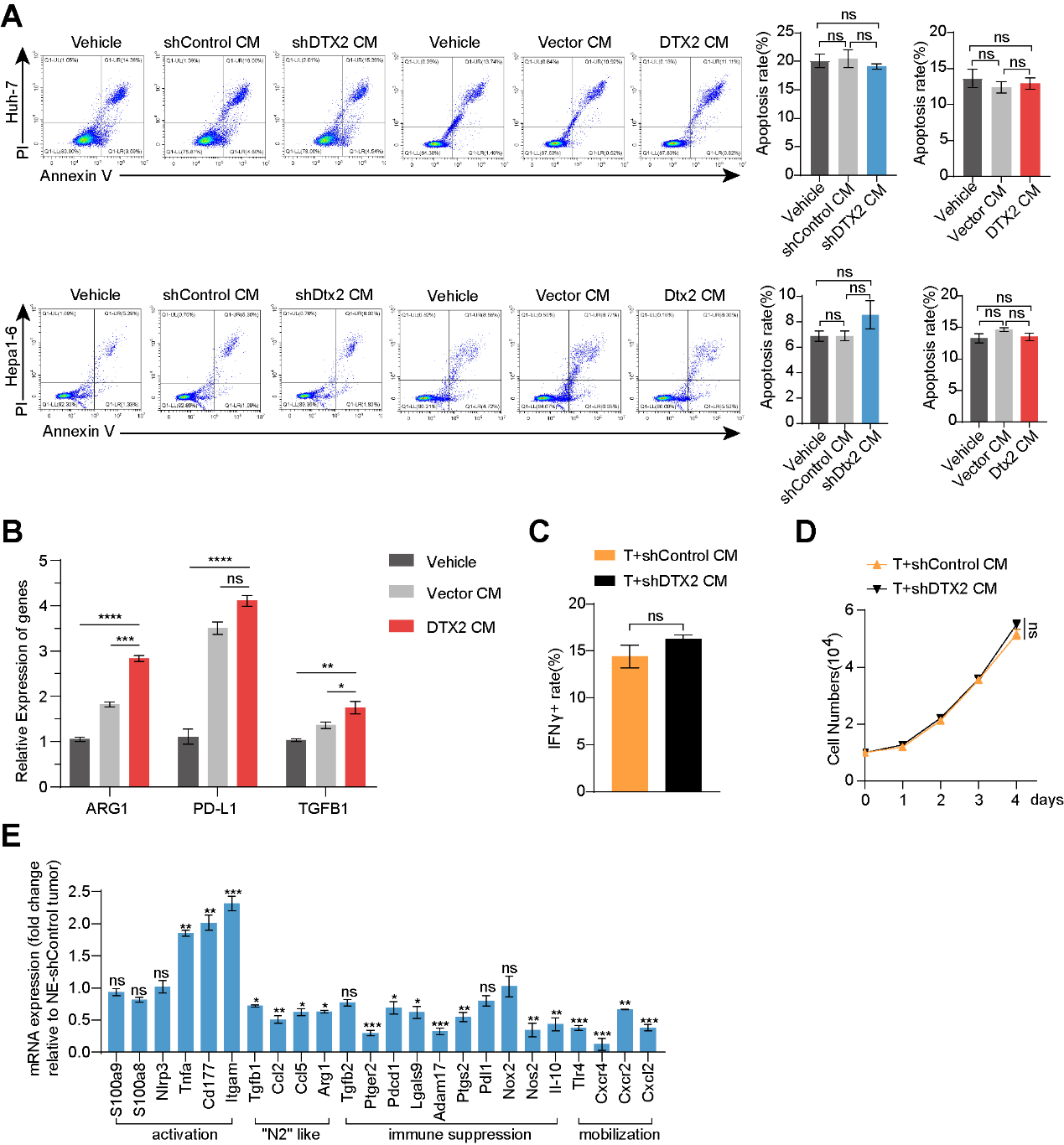


**Supplementary Figure S8. DTX2 in HCC** **promotes neutrophil chemotaxis and neutrophil polarization towards pro-tumor phenotype.**

(A) The apoptosis level of neutrophils detected by flow cytometry. (B) The qPCR detection of migrated neutrophils attracted by different groups of culture medium. (C) IFNγ expression level of CD8+ T cells cultured with different groups of culture medium from HCC cells detected by flow cytometry. (D) Cell counting number of CD8+ T cells cultured with different groups of culture medium from HCC cells. (E) Phenotypic markers of TANs isolated from subcutaneous tumors constructed by Hepa1-6 shControl cells or Hepa1-6 shDTX2 cells detected by qPCR. Data are presented as the mean ± SDs. *p < 0.05, **p < 0.01, ***p < 0.001. ns, nonsignificant difference.


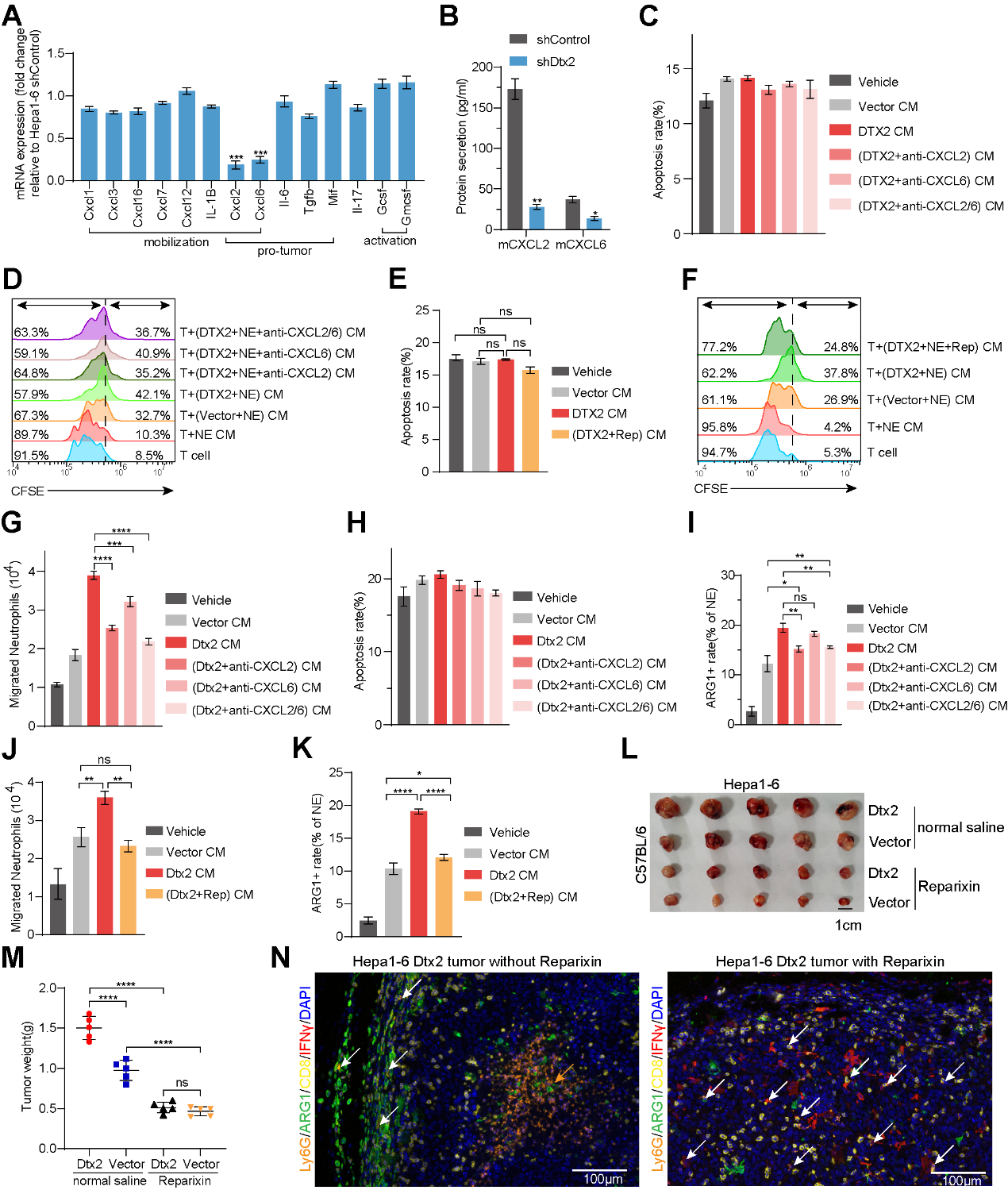


**Supplementary Figure S9. Targeting CXCL2/CXCL6-CXCR1/CXCR2 axis weaken the effect of DTX2 on neutrophils and CD8+ T cells and inhibit tumor growth.**

(A) The expression levels of cytokines or chemokines detected by qPCR. (B) The secretion levels of mouse CXCL2 and mouse CXCL6 detected by ELISA. (C) The apoptosis level of neutrophils detected through flow cytometry. (D) CFSE staining of CD8+ T cells. The dotted line is the CFSE staining peak of non-proliferating cells. (E) Reparixin was added to the culture medium of Huh-7 with DTX2 overexpression. The apoptosis level of neutrophils was detected. (F) CFSE staining of indicated groups of CD8+ T cells detected by flow cytometry. The dotted line is the CFSE staining peak of non-proliferating cells. (G) Neutrophil migration assays using indicated groups of medium. (H) ARG1 expression level of mouse neutrophils measured by flow cytometry. The dotted line indicates the boundary between ARG1- and ARG1+. (I) IFNγ expression level of mouse CD8+ T cells detected through flow cytometry. (J) Reparixin was added to the culture medium of Hepa1-6 with Dtx2 overexpression. Neutrophil chemotaxis was detected through neutrophil migration assay. (K) ARG1 expression level of mouse neutrophils measured by flow cytometry. (L-M) The tumor image (L) and tumor burden (M) of subcutaneous tumors (n=5 per group). (N) Multiplex immunofluorescence staining of subcutaneous tumors constructed by Hepa1-6 with Dtx2 overexpression cells with or without reparixin treatment. Data are presented as the mean ± SDs. *p < 0.05, **p < 0.01, ***p < 0.001，****p < 0.0001. NE, neutrophil; Rep, reparixin; ns, nonsignificant difference.


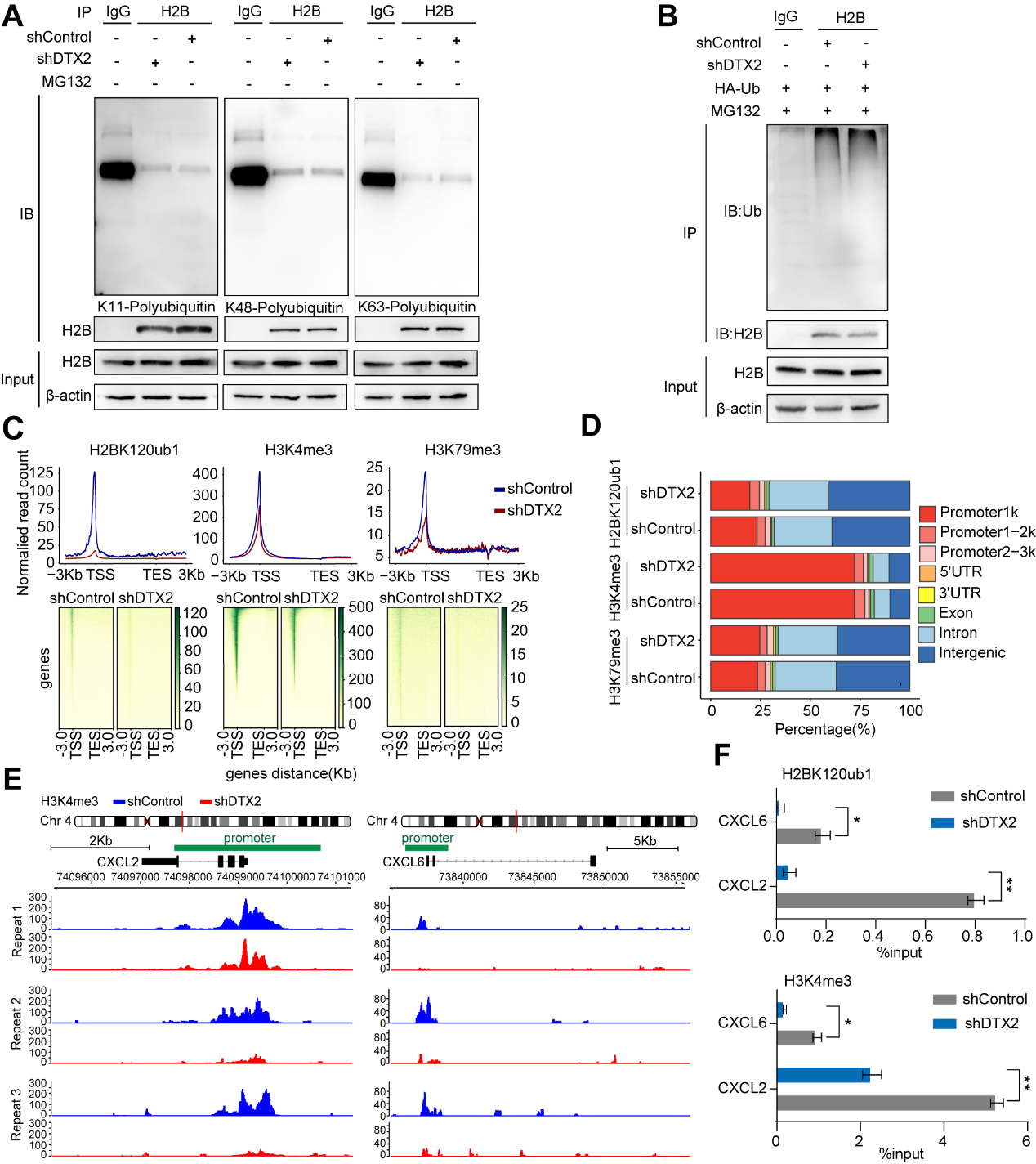


**Supplementary Figure S10. DTX2 affects H2B-Ub and the histone epigenetic modification levels of CXCL2 and CXCL6.**

(A) K11-Polybiquitin modification, K48-Polybiquitin modification and K63-Polybiquitin modification of H2B in Huh-7 shControl cells and Huh-7 shDTX2 cells. (B) Proteasome related polyubiquitin modification of H2B in Huh-7 shControl cells and Huh-7 shDTX2 cells treated with MG132. (C) Peak signal distribution in genome and peak signal distribution heat map in genome of CUT&TAG using H2B-Ub, H3K4me3 and H3K79me3 antibody. (D) Statistical map of peak signal distribution in genome of CUT&TAG using H2B-Ub, H3K4me3 and H3K79me3 antibody. (E) IGV diagram based on the triple replication experiment results of CUT&Tag obtained using anti-H3K4me3 at the CXCL2 and CXCL6 genomic. (F) ChIP-qPCR of H2BK120ub1 and H3K4me3 in the peak distribution regions of CXCL2 gene and CXCL6 gene. Data are presented as the mean ± SDs. *p < 0.05, **p < 0.01. Ub, ubiquitin.


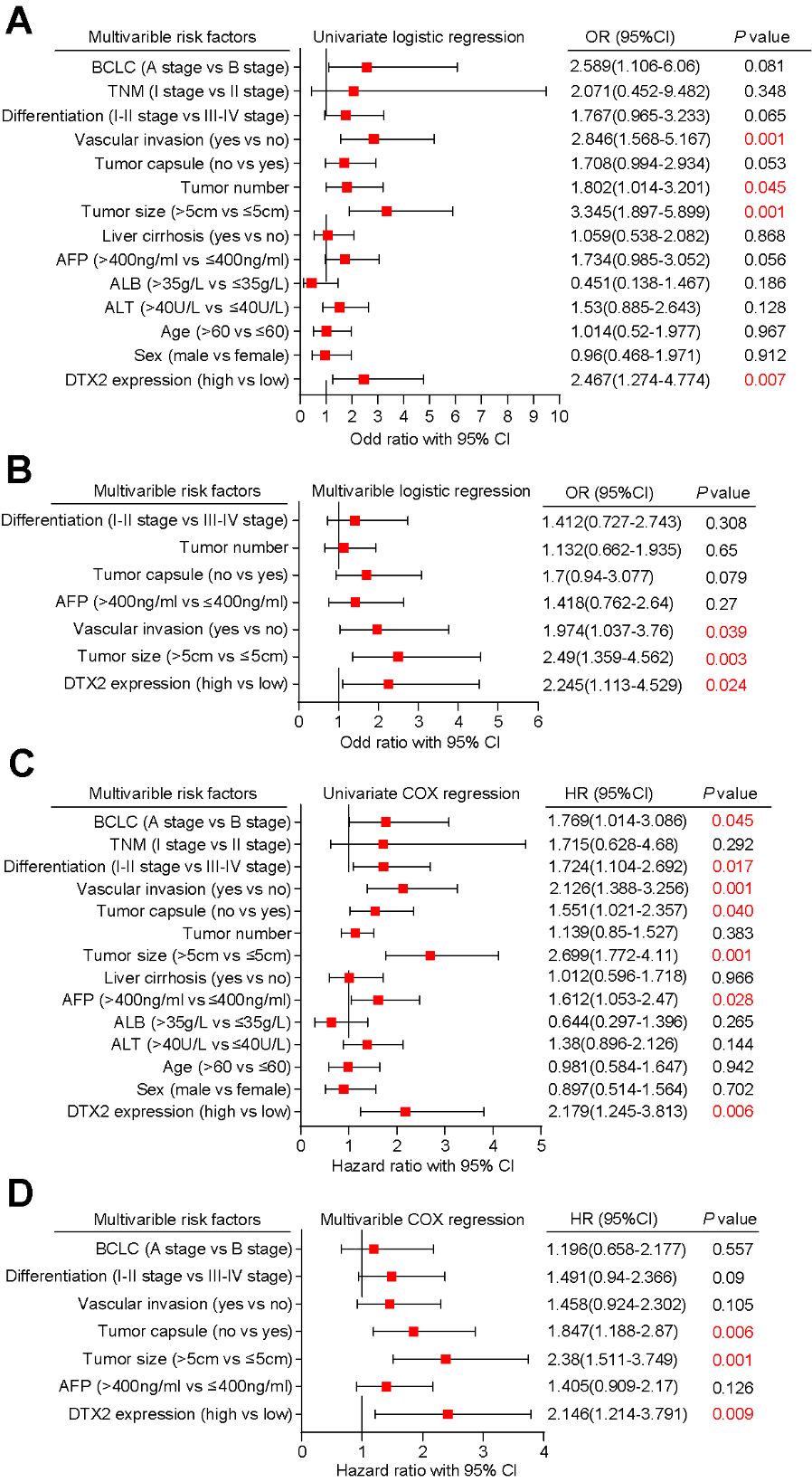


**Supplementary Figure S11. DTX2 suggests poor prognosis for HCC patients**

(A-B) Univariate logistic regression analysis (A) and multivariate logistic regression analysis (B) of clinical data from HCC patients. (C-D) Univariate COX regression analysis (C) and multivariate COX regression analysis (D) of clinical data from HCC patients. BCLC, Barcelona Clinic Liver Cancer; TNM, tumor node metastasis classification; AFP, alpha-fetoprotein; ALT, alanine aminotransferase; ALB, albumin; CI, confidence intervals.


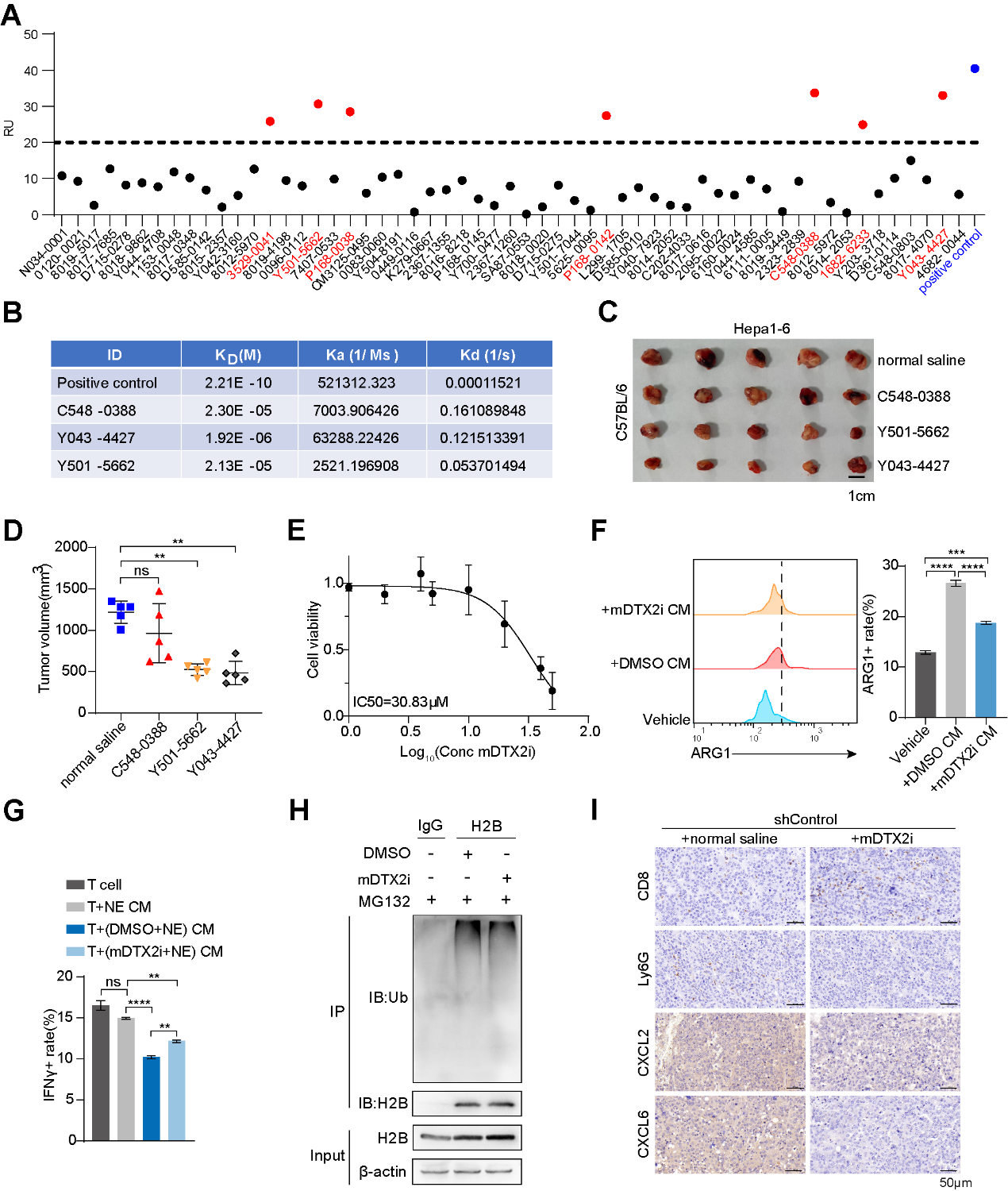


**Supplementary Figure S12. Screening and validation of mouse DTX2 protein inhibitor**

(A) The affinity of 57 small molecule compounds and positive reference antibody against mouse DTX2 protein detected by SPR. (B) The equilibrium dissociation constant (K_D_), association rate constant (Ka), and dissociation rate constant (Kd) of positive control or compounds against mouse DTX2 protein. (C-D) the tumor image (C) and tumor burden (D) of subcutaneous tumors (n=5 per group). (E) IC50 of Y501-5662 (selected as mDTX2i) detected in Hepa1-6. (F) ARG1 expression level of mouse neutrophils measured by flow cytometry. The dotted line indicates the boundary between ARG1- and ARG1+. (G) mDTX2i was added to the culture medium of Hepa1-6 and neutrophils. IFNγ expression level of mouse CD8+ T cells was detected through flow cytometry. (H) Proteasome related polyubiquitin modification of H2B in Hepa1-6 cells treated with MG132 and mDTX2i. (I) IHC staining of Ly6G, CD8, CXCL2 and CXCL6 in subcutaneous tumors treated with normal saline or mDTX2i respectively. Data are presented as the mean ± SDs. **p < 0.01, ***p < 0.001, ****p < 0.0001. RU, Relative unit; IC50, 50% inhibitory concentration; Conc, concentration; mDTX2i, mouse DTX2 inhibitor.


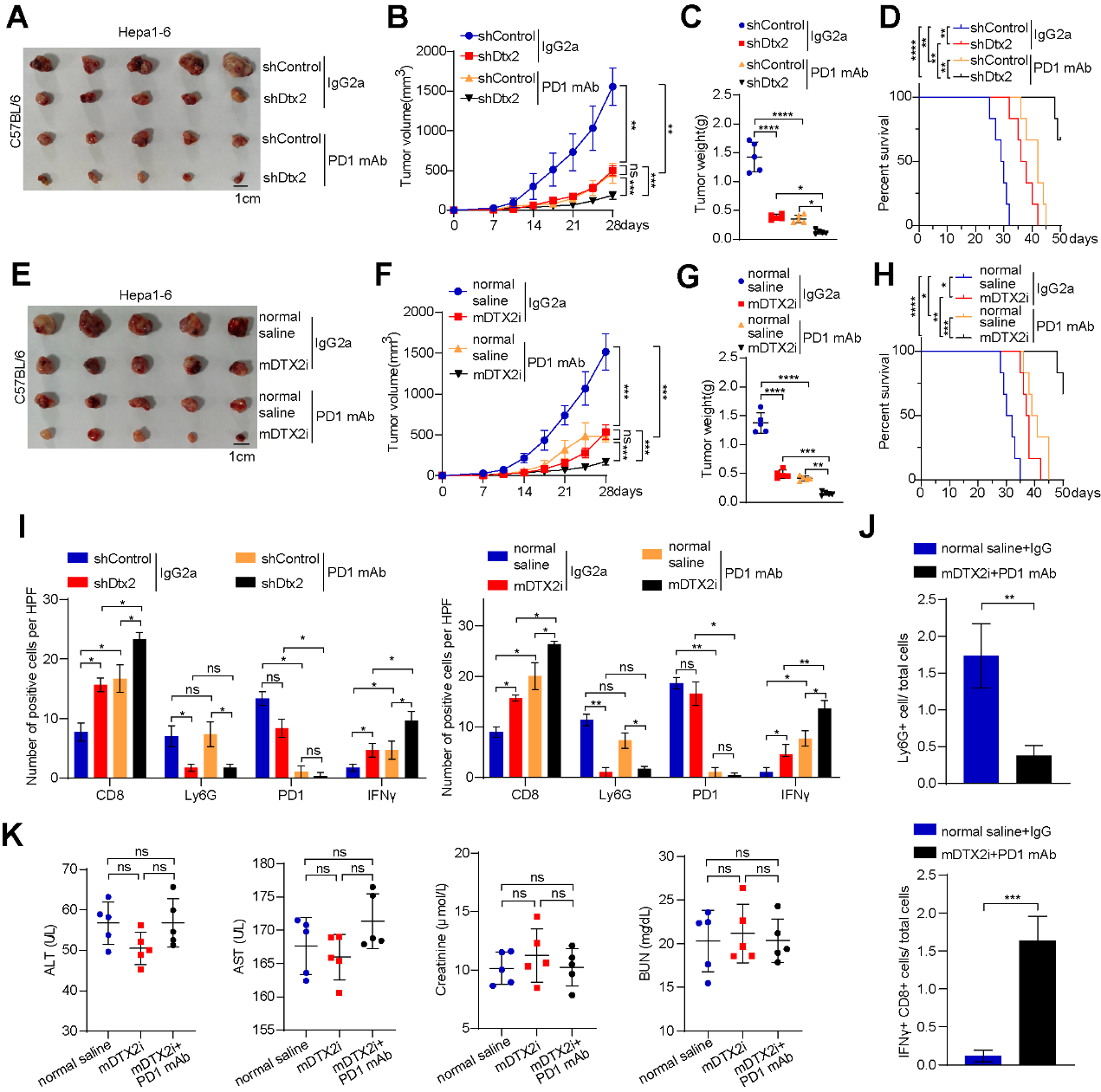


**Supplementary Figure S13. Targeting DTX2 sensitizes the efficacy of PD1 antibody on HCC**

(A-D) Tumor image (A), tumor growth curve (B), tumor burden (C) and mice survival curve (D) of subcutaneous tumors constructed by Hepa1-6 shControl and Hepa1-6 shDtx2 cells treated with IgG2a or PD1 antibody respectively (n=5 per group). (E-H) Tumor image (E), tumor growth curve (F), tumor burden (G) and mice survival curve (H) of subcutaneous tumors constructed by Hepa1-6 treated with IgG2a, PD1 antibody, saline, or mDTX2i (n=5 per group). (I) Statistical chart of CD8, Ly6G, PD1 and IFNγ staining positive cells in different groups of subcutaneous tumors. (J) Statistical graph of CD8+ IFNγ+ cell percentage and Ly6G+ cell percentage in multiplex immunofluorescence staining of the orthotopic tumors. (K) Statistical graph of liver function and renal function detection in mice with orthotopic tumor. Data are presented as the mean ± SDs. **p < 0.01, ***p < 0.001, ****p < 0.0001. mAb, mouse antibody.


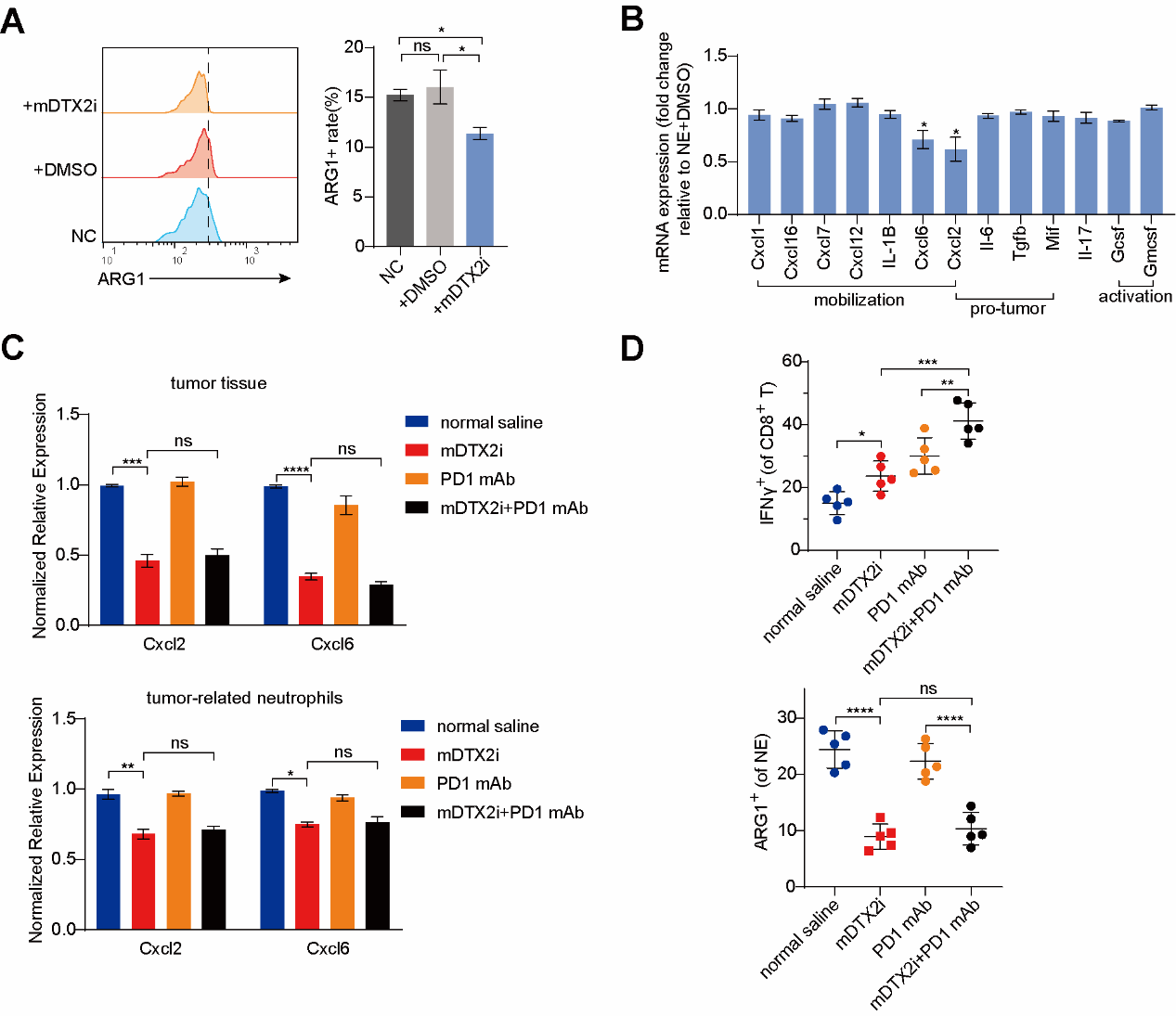


**Supplementary Figure S14. mDTX2i weakens pro-tumoral neutrophil polarization in vitro.**

(A) mDXT2i was added to the culture medium of neutrophils alone for 12h. ARG1 expression level in neutrophils was measured by flow cytometry. The dotted line indicates the boundary between ARG1- and ARG1+ cells. (B) mDXT2i was added to the culture medium of neutrophils alone for 12h. The expression levels of neutrophil-related cytokines and chemokines were measured by qPCR. (C) The qPCR detection of Cxcl2 and Cxcl6 in orthotopic tumor tissue or tumor-related neutrophils sorted by flow cytometry. (D) Proportions of ARG1+ cells among total neutrophils and IFN+ CD8+ T cells among total CD8+ T cells. Data are presented as the mean ± SDs. *p < 0.05, **p < 0.01, ***p < 0.001, ****p < 0.0001.

**Supplementary Tables**

**Table S1. Sequences of primers**

| **Primer names** | **Sequence (5'-3')** |
| --- | --- |
| Human 18s rRNA-F | CAGCCACCCGATTGAGCA |
| Human 18s rRNA-R | TAGTAGCGACGGGCGGTGTG |
| Human DTX2-F | GGGAAAGATGGAGGTATTACGG |
| Human DTX2-R | AAACCCTCTGGCAGTGAAC |
| Mouse Dtx2-F | AAGTCACAGCTCAGTTAAGAGG |
| Mouse Dtx2-R | ACAGCCAGTTTCTCCATACAG |
| Mouse β actin-F | ACCTTCTACAATGAGCTGCG |
| Mouse β actin-R | CTGGATGGCTACGTACATGG |
| Human S100A9-F | GGTCATAGAACACATCATGGAGG |
| Human S100A9-R | GGCCTGGCTTATGGTGGTG |
| Human S100A8-F | ATGCCGTCTACAGGGATGAC |
| Human S100A8-R | ACTGAGGACACTCGGTCTCTA |
| Human NLRP3-F | GATCTTCGCTGCGATCAACAG |
| Human NLRP3-R | CGTGCATTATCTGAACCCCAC |
| Human TNFA-F | CCTCTCTCTAATCAGCCCTCTG |
| Human TNFA -R | GAGGACCTGGGAGTAGATGAG |
| Human CD177-F | ATGAGCGCGGTATTACTGCTG |
| Human CD177-R | GGTCGGACACCTTCCACAC |
| Human ITGAM-F | GCCTTGACCTTATGTCATGGG |
| Human ITGAM-R | CCTGTGCTGTAGTCGCACT |
| Human CCL5-F | CCAGCAGTCGTCTTTGTCAC |
| Human CCL5-R | CTCTGGGTTGGCACACACTT |
| Human TGFB2-F | GCAGATCCTGAGCAAGCTG |
| Human TGFB2-R | GTAGGGTCTGTAGAAAGTGG |
| Human ARG1-F | GTGGAAACTTGCATGGACAAC |
| Human ARG1-R | AATCCTGGCACATCGGGAATC |
| Human PTGER2-F | CGATGCTCATGCTCTTCGC |
| Human PTGER2-R | GGGAGACTGCATAGATGACAGG |
| Human TGFB1-F | GGCCAGATCCTGTCCAAGC |
| Human TGFB1-R | GTGGGTTTCCACCATTAGCAC |
| Human PDCD1-F | CCAGGATGGTTCTTAGACTCCC |
| Human PDCD1-R | TTTAGCACGAAGCTCTCCGAT |
| Human LGALS9-F | TCTGGGACTATTCAAGGAGGTC |
| Human LGALS9-R | CCACTGGAGCTGAGAACGG |
| Human ADAM17-F | GTGGATGGTAAAAACGAAAGCG |
| Human ADAM17 -R | GGCTAGAACCCTAGAGTCAGG |
| Human PTGS2-F | CTGGCGCTCAGCCATACAG |
| Human PTGS2-R | CGCACTTATACTGGTCAAATCCC |
| Human PDL1-F | TGGCATTTGCTGAACGCATTT |
| Human PDL1-R | TGCAGCCAGGTCTAATTGTTTT |
| Human NOX2-F | TGCCAGTCTGTCGAAATCTGC |
| Human NOX2 -R | ACTCGGGCATTCACACACC |
| Human NOS2-F | TTCAGTATCACAACCTCAGCAAG |
| Human NOS2-R | TGGACCTGCAAGTTAAAATCCC |
| Human IL10-F | TCAAGGCGCATGTGAACTCC |
| Human IL10-R | GATGTCAAACTCACTCATGGCT |
| Human TLR4-F | AGACCTGTCCCTGAACCCTAT |
| Human TLR4-R | CGATGGACTTCTAAACCAGCCA |
| Human CXCR4-F | CGATGGACTTCTAAACCAGCCA |
| Human CXCR4-R | CCCACAATGCCAGTTAAGAAGA |
| Human CXCR2-F | CCTGTCTTACTTTTCCGAAGGAC |
| Human CXCR2-R | TTGCTGTATTGTTGCCCATGT |
| Mouse S100a9-F | GCACAGTTGGCAACCTTTATG |
| Mouse S100a9-R | TGATTGTCCTGGTTTGTGTCC |
| Mouse S100a8-F | AAATCACCATGCCCTCTACAAG |
| Mouse S100a8-R | CCCACTTTTATCACCATCGCAA |
| Mouse Nlrp3-F | ATTACCCGCCCGAGAAAGG |
| Mouse Nlrp3-R | TCGCAGCAAAGATCCACACAG |
| Mouse Alox5-F | GGGCTGTAGCGAGAAGCATC |
| Mouse Alox5-R | CACGGTGACATCGTAGGAGT |
| Mouse Tnfa-F | CAGGCGGTGCCTATGTCTC |
| Mouse Tnfa-R | CGATCACCCCGAAGTTCAGTAG |
| Mouse Cd177-F | GAGGGTTGCCAAGACTTGATAA |
| Mouse Cd177-R | TGCTGTTCACATCATTGCAGAG |
| Mouse Itgam-F | CCATGACCTTCCAAGAGAATGC |
| Mouse Itgam-R | ACCGGCTTGTGCTGTAGTC |
| Mouse Ccl5-F | GCTGCTTTGCCTACCTCTCC |
| Mouse Ccl5-R | TCGAGTGACAAACACGACTGC |
| Mouse Tgfb2-F | CTTCGACGTGACAGACGCT |
| Mouse Tgfb2-R | GCAGGGGCAGTGTAAACTTATT |
| Mouse Arg1-F | CTCCAAGCCAAAGTCCTTAGAG |
| Mouse Arg1-R | GGAGCTGTCATTAGGGACATCA |
| Mouse Ptger2-F | CAGCTCGGTGATGTTCTCGG |
| Mouse Ptger2-R | GAGCACCAATTCCGTTACCAG |
| Mouse Tgfb1-F | CCACCTGCAAGACCATCGAC |
| Mouse Tgfb1-R | CTGGCGAGCCTTAGTTTGGAC |
| Mouse Pdcd1-F | CAGCTTGTCCAACTGGTCG |
| Mouse Pdcd1-R | GCTCAAACCATTACAGAAGGCG |
| Mouse Lgals9-F | TTACTGGACCAATCCAAGGAGG |
| Mouse Lgals9-R | AGCTGTTCTGAAAGTTCACCAC |
| Mouse Adam17-F | ACCACTTTGGTGCCTTTCGT |
| Mouse Adam17 -R | GTCGCAGACTGTAGATCCCTT |
| Mouse Ptgs2-F | TTCCAATCCATGTCAAAACCGT |
| Mouse Ptgs2-R | AGTCCGGGTACAGTCACACTT |
| Mouse Pdl1-F | GCTCCAAAGGACTTGTACGTG |
| Mouse Pdl1-R | TGATCTGAAGGGCAGCATTTC |
| Mouse Nox2-F | AGTGCGTGTTGCTCGACAA |
| Mouse Nox2 -R | GCGGTGTGCAGTGCTATCAT |
| Mouse Nos2-F | GTTCTCAGCCCAACAATACAAGA |
| Mouse Nos2-R | GTGGACGGGTCGATGTCAC |
| Mouse Il10-F | CTTACTGACTGGCATGAGGATCA |
| Mouse Il10-R | GCAGCTCTAGGAGCATGTGG |
| Mouse Tlr4-F | ATGGCATGGCTTACACCACC |
| Mouse Tlr4-R | GAGGCCAATTTTGTCTCCACA |
| Mouse Cxcr4-F | GACTGGCATAGTCGGCAATG |
| Mouse Cxcr4-R | AGAAGGGGAGTGTGATGACAAA |
| Mouse Cxcr2-F | ATGCCCTCTATTCTGCCAGAT |
| Mouse Cxcr2-R | GTGCTCCGGTTGTATAAGATGAC |
| Human CXCL1-F | ACCCAAACCGAAGTCATAGCC |
| Human CXCL1-R | TTGTCAGAAGCCAGCGTTCA |
| Human CXCL2-F | CAGGCTACAGGGGCTGTTGT |
| Human CXCL2-R | ACATCAGGTACGATCCAGGC |
| Human CXCL3-F | CCCAGACAGAAGTCATAGCCA |
| Human CXCL3-R | ACACATCCAGACACCGTTGG |
| Human CXCL4-F | GCCAGCGCTGAAGCTGAA |
| Human CXCL4-R | GCCAACATGTAACACCAAGCA |
| Human CXCL5-F | AGCTGCGTTGCGTTTGTTTAC |
| Human CXCL5-R | TGGCGAACACTTGCAGATTAC |
| Human CXCL6-F | AGAGCTGCGTTGCACTTGTT |
| Human CXCL6-R | GCAGTTTACCAATCGTTTTGGGG |
| Human CXCL7-F | TTGTAGGCAGCAACTCACCC |
| Human CXCL7-R | TGCAAGGCATGAAGTGGTCT |
| Human CXCL8-F | TTTTGCCAAGGAGTGCTAAAGA |
| Human CXCL8-R | AACCCTCTGCACCCAGTTTTC |
| Human CXCL12-F | ATTCTCAACACTCCAAACTGTGC |
| Human CXCL12-R | ACTTTAGCTTCGGGTCAATGC |
| Human IL-6-F | ACTCACCTCTTCAGAACGAATTG |
| Human IL-6-R | CCATCTTTGGAAGGTTCAGGTTG |
| Human IL-17-F | AGATTACTACAACCGATCCACCT |
| Human IL-17-R | GGGGACAGAGTTCATGTGGTA |
| Human GCSF-F | GCTGCTTGAGCCAACTCCATA |
| Human GCSF-R | GAACGCGGTACGACACCTC |
| Human GMCSF-F | TCCTGAACCTGAGTAGAGACAC |
| Human GMCSF -R | TGCTGCTTGTAGTGGCTGG |
| Human MIF-F | GTGGTGTCCGAGAAGTCAGG |
| Human MIF-R | TTGCTGTAGGAGCGGTTCTG |
| Human CXCL16-F | GACATGCTTACTCGGGGATTG |
| Human CXCL16-R | GGACAGTGATCCTACTGGGAG |
| Human IL1B-F | ATGATGGCTTATTACAGTGGCAA |
| Human IL1B -R | GTCGGAGATTCGTAGCTGGA |
| Mouse Cxcl1-F | CTGGGATTCACCTCAAGAACATC |
| Mouse Cxcl1-R | CAGGGTCAAGGCAAGCCTC |
| Mouse Cxcl2-F | CCAACCACCAGGCTACAGG |
| Mouse Cxcl2-R | GCGTCACACTCAAGCTCTG |
| Mouse Cxcl3-F | ACACCCTACCAAGGGTTGATTTT |
| Mouse Cxcl3-R | GACTTCTGTCTGGGTGCAGTG |
| Mouse Cxcl6(Gcp2)-F | GTTCCATCTCGCCATTCATGC |
| Mouse Cxcl6(Gcp2)-R | GCGGCTATGACTGAGGAAGG |
| Mouse Cxcl7-F | CTCAGACCTACATCGTCCTGC |
| Mouse Cxcl7-R | GTGGCTATCACTTCCACATCAG |
| Mouse Cxcl12-F | TGCATCAGTGACGGTAAACCA |
| Mouse Cxcl12-R | TTCTTCAGCCGTGCAACAATC |
| Mouse Il-6-F | CTGCAAGAGACTTCCATCCAG |
| Mouse Il-6-R | AGTGGTATAGACAGGTCTGTTGG |
| Mouse Il-17-F | TCAGCGTGTCCAAACACTGAG |
| Mouse Il-17-R | CGCCAAGGGAGTTAAAGACTT |
| Mouse Gcsf-F | ATGGCTCAACTTTCTGCCCAG |
| Mouse Gcsf-R | CTGACAGTGACCAGGGGAAC |
| Mouse Gmcsf-F | GGCCTTGGAAGCATGTAGAGG |
| Mouse Gmcsf -R | GGAGAACTCGTTAGAGACGACTT |
| Mouse Mif-F | GAGGGGTTTCTGTCGGAGC |
| Mouse Mif-R | GTTCGTGCCGCTAAAAGTCA |
| Mouse Cxcl16-F | CCTTGTCTCTTGCGTTCTTCC |
| Mouse Cxcl16-R | TCCAAAGTACCCTGCGGTATC |
| Mouse Il1b-F | GAAATGCCACCTTTTGACAGTG |
| Mouse Il1b-R | TGGATGCTCTCATCAGGACAG |

**Table S2. Sequences of siRNAs and shRNAs**

| **siRNA names** | **Sequence (5'-3')** | |
| --- | --- | --- |
| siControl | | TTCTCCGAACGTGTCACGTAA |
| siDTX2 | | GCAGCTTCATCGAGCAGCAGTTTGT |
| Human-shControl-Top | | GATCCGTTCTCCGAACGTGTCACGTAATTCAAGAGATTACGTGACACGTTCGGAGAATTTTTTC |
| Human-shControl-Bottom | | AATTGAAAAAATTCTCCGAACGTGTCACGTAATCTCTTGAATTACGTGACACGTTCGGAGAACG |
| Human-shDTX2-Top1 | | GATCCGCAGCTTCATCGAGCAGCAGTTTGTCTCGAGACAAACTGCTGCTCGATGAAGCTGCTTTTTTG |
| Human- shDTX2-Bottom1 | | AATTCAAAAAAGCAGCTTCATCGAGCAGCAGTTTGTCTCGAGACAAACTGCTGCTCGATGAAGCTGCG |
| Human-shDTX2-Top2 | | GATCCGAAGTCTGCAGTGTCCCTCCTGCAAACTCGAGTTTGCAGGAGGGACACTGCAGACTTTTTTTTG |
| Human- shDTX2-Bottom2 | | AATTCAAAAAAAAGTCTGCAGTGTCCCTCCTGCAAACTCGAGTTTGCAGGAGGGACACTGCAGACTTCG |
| Mouse-shControl-Top | | GATCCGTTCTCCGAACGTGTCACGTAATTCAAGAGATTACGTGACACGTTCGGAGAATTTTTTC |
| Mouse-shControl-Bottom | | AATTGAAAAAATTCTCCGAACGTGTCACGTAATCTCTTGAATTACGTGACACGTTCGGAGAACG |
| Mouse-shDtx2-Top1 | | GATCCGGGTATAACTATACTGTCAACTATGTTCAAGAGACATAGTTGACAGTATAGTTATACCCTTTTTTG |
| Mouse-shDtx2-Bottom1 | | AATTCAAAAAAGGGTATAACTATACTGTCAACTATGTCTCTTGAACATAGTTGACAGTATAGTTATACCCG |
| Mouse-shDtx2-Top2 | | GATCCGCCCATACAATAAACCTTCACTGTCTTTCAAGAGAAGACAGTGAAGGTTTATTGTATGGGTTTTTTG |
| Mouse-shDtx2-Bottom2 | | AATTCAAAAAACCCATACAATAAACCTTCACTGTCTTCTCTTGAAAGACAGTGAAGGTTTATTGTATGGGCG |
| Mouse-shDtx2-Top3 | | GATCCGCCGGAGCAGGTGATCAGAAAGTACATTCAAGAGATGTACTTTCTGATCACCTGCTCCGGTTTTTTG |
| Mouse-shDtx2-Bottom3 | | AATTCAAAAAACCGGAGCAGGTGATCAGAAAGTACATCTCTTGAATGTACTTTCTGATCACCTGCTCCGGCG |

**Table S3. Sequences of primers used in ChIP-qPCR**

| **Primer names** |  | **Sequence (5'-3')** |
| --- | --- | --- |
| hCXCL2 (chr4:74098780- 74099313) for H3K4me3 | F | GCGATGGGCGAGACTTACAT |
|  | R | CATCGCCTTCCTTCCGAACT |
| hCXCL2 (chr4:74097674- chr4:74097846) for H2B-Ub | F | ACCTACTCAGGGCACCCAT |
|  | R | GCTCAAACACATTAGGCGCAA |
| hCXCL6 (chr4:73835876- chr4:73836101) for H3K4me3 | F | AGCTCCTTTATGCAGGCTCC |
|  | R | CCGGGAGAGCTGTTTGATGT |
| hCXCL6 (chr4:73837732-chr4: 73837882) for H2B-Ub | F | GCATCCTAGGGTAAAGAGTAACA |
|  | R | CCGAAAAGGCTGTGGATTTC |

**Table S4. Antibodies used for Western-blot, IHC, IF, co-ip, neutralization and intraperitoneal injection of mice in this study**

| **Antibody** | **WB** | **IHC/IF** | **Co-ip** | **Species** | **Neut** | **IP injection** | **Co.** |
| --- | --- | --- | --- | --- | --- | --- | --- |
| DTX2 (orb156649) | 1:1000 | 1:100 | 1.0μg | Rabbit |  |  | Biorbyt |
| β-actin (AC026) | 1:10000 |  |  | Rabbit |  |  | ABclonal |
| CD8 (orb323288) |  | 1:100 |  | Rabbit |  |  | Biorbyt |
| CD4 (orb4830) |  | 1:100 |  | Rabbit |  |  | Biorbyt |
| Ly6G (orb44840) |  | 1:100 |  | Mouse |  |  | Biorbyt |
| CD8a (BE0061) |  |  |  | Mouse |  | 100μg | Bioxcell |
| CD4 (BE0003-1) |  |  |  | Mouse |  | 100μg | Bioxcell |
| Ly6G (BE0075-1) |  |  |  | Mouse |  | 100μg | Bioxcell |
| IgG2a (BE0085) |  |  |  | Mouse |  | 100μg | Bioxcell |
| CXCL2 (MAB452) |  |  |  | E. coli | 50ng/mL |  | R&D Systems |
| CXCL2 (A16108E) |  |  |  | Mouse | 50ng/mL |  | BioLegend |
| CXCL2 (16325-1-AP) |  | 1:100 |  | Rabbit |  |  | Proteintech |
| CXCL6 (A20098E) |  |  |  | Rat | 50ng/mL |  | BioLegend |
| CXCL6 (MAB333) |  |  |  | E. coli | 50ng/mL |  | R&D Systems |
| CXCL6 (orb638448) |  | 1:100 |  | Rabbit |  |  | Biorbyt |
| IgG (30000-0-AP) |  |  | 1.0μg | Rabbit |  |  | Proteintech |
| H2B (#12364) | 1:1000 |  | 1.0μg | Rabbit |  |  | CST |
| Flag (AE005) |  |  | 1.0μg | Mouse |  |  | ABclonal |
| Flag (AE063) | 1:1000 |  |  | Rabbit |  |  | ABclonal |
| K11-specific Polyubiquitin (orb761788) | 1:1000 |  |  | Rabbit |  |  | Biorbyt |
| K48-specific Polyubiquitin (#8081) | 1:1000 |  |  | Rabbit |  |  | CST |
| K63-specific Polyubiquitin (#5621) | 1:1000 |  |  | Rabbit |  |  | CST |
| Ubiquitin (#3936) | 1:1000 |  |  | Mouse |  |  | CST |
| H2BK120ub1 (#5546) | 1:1000 |  |  | Rabbit |  |  | CST |
| H3K4me3 (#9751) | 1:1000 |  |  | Rabbit |  |  | CST |
| H3K79me3 (#74073) | 1:1000 |  |  | Rabbit |  |  | CST |
| PD1 (18106-1-AP) |  | 1:100 |  | Rabbit |  |  | Proteintech |
| PD1 (BE0146) |  |  |  | Mouse |  | 100μg | Bioxcell |
| IFNγ (15365-1-AP) |  | 1:100 |  | Rabbit |  |  | Proteintech |
| CD66B (ab300122) |  | 1:100 |  | Rabbit |  |  | Abcam |

**Table S5. Antibodies used for flow cytometry**

| **Name** | **Co.** | **Cat Num** |
| --- | --- | --- |
| Anti-CD45 | BioLenged | 103116 |
| Anti-CD3 | BioLenged | 100214 |
| Anti-CD4 | BioLenged | 100434 |
| Anti-CD8 | BioLenged | 100706 |
| Anti-B220 | BioLenged | 103228 |
| Anti-NK1.1 | Invitrogen | 17-5941-82 |
| Anti-CD25 | BioLenged | 101904 |
| Anti-Foxp3 | Invitrogen | 48-5773-82 |
| Anti-PD-1 | BioLenged | 135218 |
| Anti-INFγ | BioLenged | 505826 |
| Anti-Granzyme B | BioLenged | 372206 |
| Anti-CD11b | Invitrogen | 11-0112-85 |
| Anti-CD11c | Invitrogen | 12-0114-81 |
| Anti-MHC-II | Invitrogen | 48-5321-82 |
| Anti-Ly6G | BioLenged | 127618 |
| Anti-F4/80 | Invitrogen | 17-4801-82 |
| Anti-Ly6C | BioLenged | 128010 |
| Anti-TNFa | Invitrogen | 48-7349-42 |
| Anti-ARG1 | Invitrogen | 48-3697-82 |
| Anti-CD206 | Invitrogen | 47-2061-82 |

**Table S6. Antibodies used for CyTOF**

| **Name** | **Co.** | **Lable** | **Cat Num** |
| --- | --- | --- | --- |
| anti-CD45 | BioLegend | 89Y | 103102 |
| anti-CD3ε | BioLegend | 115ln | 100302 |
| anti-CD49b | BioLegend | 141Pr | 108902 |
| anti-CD172a | BioLegend | 142Nd | 144002 |
| anti-Gr-1 | BioLegend | 143Nd | 108402 |
| anti-CD183 | BioLegend | 144Nd | 126502 |
| anti-CD161 | BioLegend | 145Nd | 108702 |
| anti-CD279 | BioLegend | 146Nd | 135202 |
| anti-Ly6G | BioLegend | 147Sm | 127602 |
| anti-Ly6C | eBioscience | 148Nd | 128002 |
| anti-CD64 | BioLegend | 149Sm | 139302 |
| anti-CD25 | BioLegend | 150Nd | 101902 |
| anti-CD62L | BioLegend | 151Eu | 104402 |
| anti-CD19 | BioLegend | 152Sm | 115502 |
| anti-CD44 | BioLegend | 153Eu | 103002 |
| anti-Ki67 | eBioscience | 154Sm | 14-5698-82 |
| anti-CD103 | BioLegend | 155Gd | 121402 |
| anti-CD194 | BioLegend | 156Gd | 131202 |
| anti-CD366 | BioLegend | 157Gd | 119702 |
| anti-CD45R | BioLegend | 158Gd | 103202 |
| anti-F4/80 | Biorad | 159Tb | MCA497G |
| anti-CD206 | BioLegend | 160Gd | 141702 |
| anti-iNOS | eBioscience | 161Dy | 14-5920-82 |
| anti-FOXP3 | eBioscience | 162Dy | 14-5773-82 |
| anti-MERTK | BioLegend | 163Dy | 151502 |
| anti-CD11c | BioLegend | 164Dy | 117302 |
| anti-CD278 | BioLegend | 165Ho | 313502 |
| anti-CD192 | R&D systems | 166Er | MAB55381-100 |
| anti-TCR β | BioLegend | 167Er | 109202 |
| anti-CD27 | BioLegend | 168Er | 124202 |
| anti-CD69 | BioLegend | 169Tm | 104502 |
| anti-T-bet | BioLegend | 170Er | 644802 |
| anti-CX3CR1 | BioLegend | 171Yb | 149002 |
| anti-CD127 | BioLegend | 172Yb | 135002 |
| anti-GranB (Fluidigm) | Fluidigm | 173Yb | 3173006B |
| anti-CD196 | BioLegend | 174Yb | 129802 |
| anti-MHC-II | BioLegend | 175Lu | 107602 |
| anti-TCR γδ | BioLegend | 176Yb | 118140 |
| anti-CD4 | BioLegend | 197Au | 100576 |
| anti-CD8a | BioLegend | 198pt | 100746 |
| anti-CD11b | BioLegend | 209Bi | 101202 |

**Reference**

1. Zunder ER, Finck R, Behbehani GK, Amir el AD, Krishnaswamy S, Gonzalez VD, et al. Palladium-based mass tag cell barcoding with a doublet-filtering scheme and single-cell deconvolution algorithm. Nat Protoc. 2015;10(2):316-33.

2. Finck R, Simonds EF, Jager A, Krishnaswamy S, Sachs K, Fantl W, et al. Normalization of mass cytometry data with bead standards. Cytometry A. 2013;83(5):483-94.

3. Samusik N, Good Z, Spitzer MH, Davis KL, Nolan GP. Automated mapping of phenotype space with single-cell data. Nat Methods. 2016;13(6):493-6.

4. van der Maaten L, Hinton G. Visualizing Data using t-SNE. J Mach Learn Res. 2008;9:2579-605.

5. Kaya-Okur HS, Wu SJ, Codomo CA, Pledger ES, Bryson TD, Henikoff JG, et al. CUT&Tag for efficient epigenomic profiling of small samples and single cells. Nat Commun. 2019;10(1):1930.
